# An Atypical Dinuclear Copper(II) 1,2,4-Triazolyl Complex as a Catalyst for Palladium-Free Csp-Csp Homocoupling of Phenylacetylene

**DOI:** 10.1021/acsorginorgau.6c00013

**Published:** 2026-04-14

**Authors:** Lorenzo Luciani, Nicola Sargentoni, Magda Monari, Rossana Galassi

**Affiliations:** † 201848 University of Camerino, School of Science and Technology, Chemistry Division @CHIP, Via Madonna delle Carceri, Camerino 62032, Italy; ‡ University of Bologna, Chemistry Department, ″Giacomo Ciamician″, Via Gobetti 85, Bologna 40129, Italy

**Keywords:** copper(II) complexes, triazole ligand, X-ray crystallography, self-aggregation, phenylacetylene, C_sp_-C_sp_ homocoupling reactions, Glaser-type reaction

## Abstract

An unusual dinuclear copper­(II) complex, labeled as [L_2_Cu_2_(solv)_2_]•2­(solv) (where solv = CH_3_CN or H_2_O and L = 3,5-bis­(trifluoroacetamido)-1,2,4-triazo-1-yl), with L acting as an O,N,N′,O′-donor chelating ligand, was prepared by reaction with Cu_2_O. The single crystal XRD analysis and the susceptibility measurements revealed a pentacoordinated dinuclear Cu­(II) complex with the two Cu atoms in a square pyramidal environment, doubly bridged by the 3,5-substituted triazolate ligands and one coordinated acetonitrile in the apical position, completing the coordination sphere. Systematic voids in the solid-state packing accommodate two additional acetonitrile molecules; all of the acetonitrile molecules can be readily replaced by water or other solvents. The new copper­(II) complex was investigated as a catalyst in the C_sp_-C_sp_ homocoupling reaction of phenylacetylene (Glaser-type reaction), yielding satisfactory results in terms of both isolated yields and procedural simplicity. For a better understanding of the catalytic results, spontaneous aggregation in solution of this copper­(II) complex was investigated. The dilution experiments in polar solvents, followed by UV–visible spectroscopy, highlighted the self-aggregation behaviors, which were further corroborated by ESI-MS analyses. The dissociative equilibrium constants, *K*_d_, were determined in ethanol, *K*_d_ = 3.89 × 10^–3^ M, and in CH_3_CN, *K*_d_ = 8.10 × 10^–2^ M, evidencing the role of the solvent in the association process.

## Graphical abstract

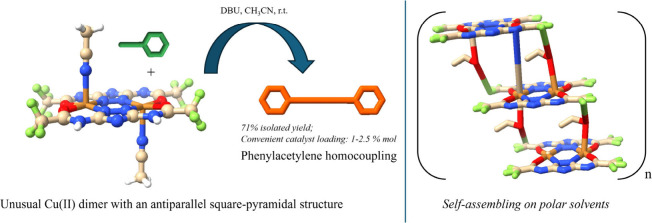

Among the azole ligands used in copper coordination chemistry, 1,2,3-triazole ligands are particularly relevant^1–3^ because of their straightforward synthesis via Copper Azide Click Cyclization (CuACC).^4^ The highly symmetrical 1,2,4-triazoles are mostly employed as bihapto N,N ligands, either in their unmodified form or with substituents at the 3 and 5 positions.^5,6^ Notably, the substituent groups at the aforementioned positions of the 1,2,4-triazole ring significantly influence the coordination properties of the triazole, affecting factors like: (i) the availability of additional coordination sites, (ii) the solubility of the resulting metal complexes, (iii) the topological characteristics,^7^ (iv) the metal nuclearity, and (v) the potential applications of the final products.^8–10^ Considering the various factors involved, copper complexes with 1,2,4-triazoles are applied as effective homogeneous catalysts.^6,11,12^ A potential area of application of copper-based catalysts is the homocoupling reactions of terminal alkynes.^13,14^ Nevertheless, the homocoupling process often entails the presence of oxidants such as iodine (I_2_)^15^ or oxygen (O_2_),^16^ as well as chelating ligands and bases,^17,18^ and may require stringent conditions, including prolonged reflux times,^19,20^ or the use of expensive Pd salts^21^ or complexes^22^ as cocatalysts or catalysts. Hence, the development of numerous strategies, including the employment of heterogeneous catalysts, such as nanoparticles,^23^ zeolites,^24^ MOFs,^20^ or complexes anchored on solid supports,^25^ or the use of ionic liquids, and supercritical carbon dioxide (sc-CO_2_) as more efficient and environmentally benign solvents^26–28^ was investigated. A critical aspect of applying copper complexes in homogeneous catalysis is the solubility of the resulting catalysts. In light of these considerations, a novel Copper­(II) catalyst was developed using a 3,5-disubstituted 1,2,4-triazole ligand, incorporating acetamido groups that provide additional sites for coordination and featuring terminal trifluoromethyl functional groups to enhance overall solubility. Ligands of similar nature, which can function as either heteroleptic N,O or homoleptic N,N ligands, yielded square planar Cu­(II) complexes, therefore leaving unoccupied binding sites on the copper center that may contribute to catalytic activity.^10,29^ The synthesis of the copper­(II) complex involves the reaction of ligand L in acetonitrile under reflux conditions utilizing solid Cu_2_O (Scheme 1). Over several hours of refluxing, the reaction mixture transitioned from colorless to green, and the green-colored solid recovered by drying the filtered solution under reduced pressure.

**Scheme 1:**
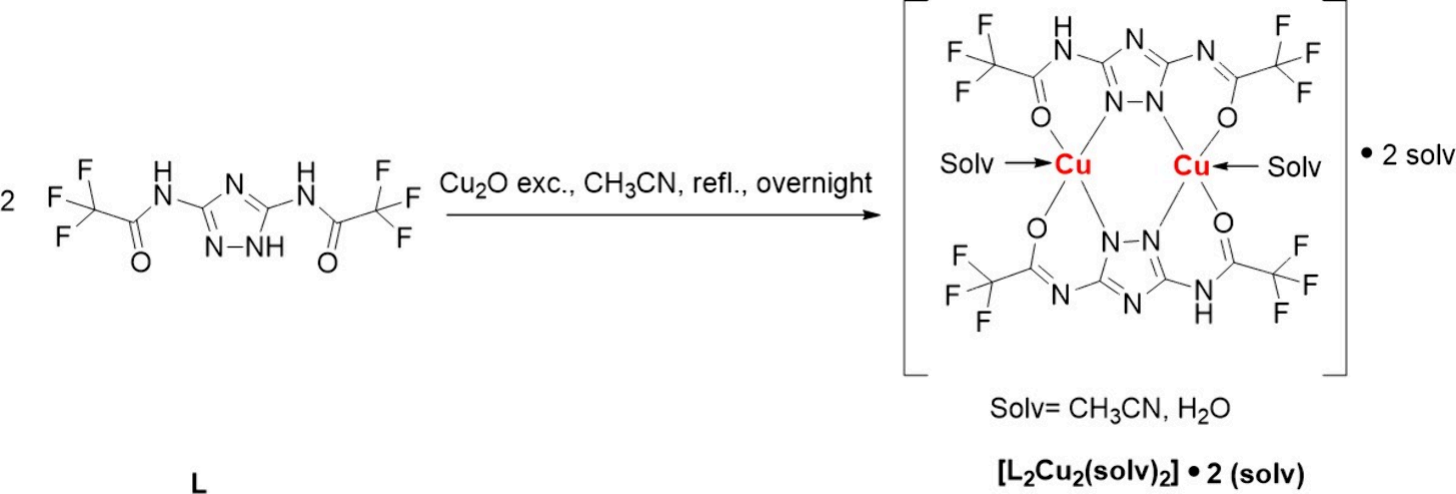
Schematic View of the Synthesis of Complex [L_2_Cu_2_(solv)_2_]•2­(solv)

Single crystals of [L_2_Cu_2_(CH_3_CN)_2_]•2­(CH_3_CN) suitable for XRD analysis were obtained after the slow evaporation of CH_3_CN solutions. The crystal structure of [L_2_Cu_2_(CH_3_CN)_2_]•2CH_3_CN (Figure 1) shows the formation of a dinuclear centrosymmetric Cu­(II) complex in which the Cu atoms are doubly N,N bridged by 1,2,4 substituted triazoles forming a planar dimetallacyclic six-membered ring [Cu1–N4 1.965(5), Cu1–N5 1.969(4) Å, respectively]. Further coordination of two oxygens from the two acetamido substituents in positions 3 and 5 of the triazoles [Cu1–O1 1.927(4) and Cu1–O2 1.949(5) Å, respectively] forms four planar six-membered metallacyclic rings. The Cu­(II) ions coordinated by the two tetradentate substituted triazole ligands are displaced out of the least-squares plane comprising the atoms of the two 3,5-bis­(trifluoroacetamido)-1,2,4-triazolate ligands by 0.15 Å. The coordination of each Cu atom is completed by the presence of one CH_3_CN ligand [Cu1–N­(acetonitrile) 2.398(6) Å] located in the apical position and pointing in opposite directions with respect to the planar framework, and the geometry around the Cu atoms is distorted square pyramidal [τ 5 = 0.06] (Figure 1, right). The free CH_3_CN solvent molecule lies almost parallel to the equatorial plane with a Cu···N distance equal to 3.492 Å, in the *trans* position to the coordinated CH_3_CN. The molecule conforms almost exactly to crystallographic symmetry (see Supporting Information Figures 1S and 2S), and the half molecule present in the asymmetric unit has an inversion center at the midpoint of the Cu–Cu vector [Cu···Cu distance 3.866(3) Å] (Figure 1, right, and Figure S1 Supporting Information). In the crystal packing, a network of nonclassical intermolecular C–H···F H-bonds involving the H atoms of the coordinated and “free” CH_3_CN groups and the fluorine atoms of the CF_3_ groups are present (Figure 1 on the right). Even though numerous structures containing Cu­(II) ions and 1,2,4 double-bridging triazoles have been reported,^6,30,31^ the molecular arrangement presented in this study is uncommon in literature. The doubly bridging substituted triazolate ligands form, through the O atoms, by chelating the Cu atoms, four chelate planar six-membered rings (Figure 1); similar arrangements has been found only in [Cu­(daat)­(NO_3_)­(H_2_O)]_2_ (data = 3,5-diacetylamino-1,2,4-triazole),^31^ and in [Cu_2_(C_6_H_5_N_3_O_4_)_2_(H_2_O)_4_]·2H_2_O.^32^ However, in the two complexes quoted above, the Cu atom displays an octahedral environment, and in the latter case, the fused rings are nonplanar.

**Figure 1:**
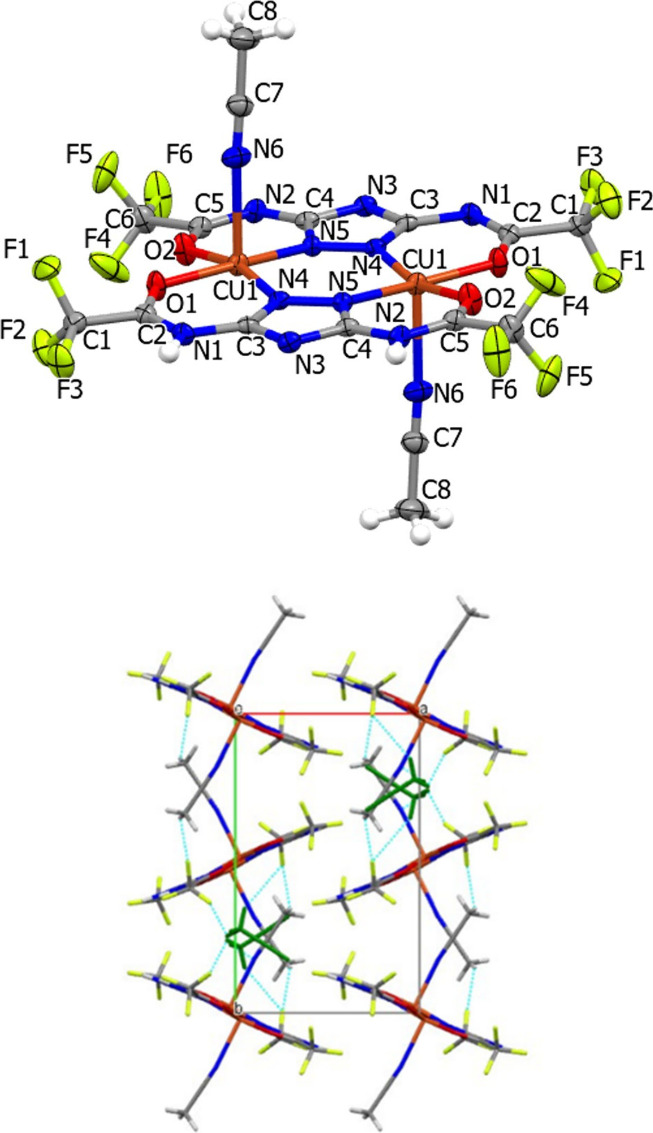
ORTEP plot at 30% of probability level of [L_2_Cu_2_(CH_3_CN)_2_] with the numbering scheme (top), and of the unit cell packing showing the network of the CH···F H bonding in [L_2_Cu_2_(CH_3_CN)_2_]•2­(CH_3_CN) (dotted pale blue lines, bottom). The uncoordinated CH_3_CN molecule is represented in dark green.

A particular feature of the presented copper­(II) compound is the ready exchange of acetonitrile molecules with water once exposed to air. The early observation was the fading of crystals when removed from the mother liquor, resulting in the formation of the copper­(II) compound containing the [Cu_2_L_2_] core and four water molecules instead of the acetonitrile ones. XRD powder diffraction patterns were recorded for freshly prepared crystals taken to dryness and compared with those both simulated and acquired for wet crystals of [L_2_Cu_2_(CH_3_CN)_2_]•2­(CH_3_CN) (Figure 2). The pattern recorded for wet crystals displays peaks at 9.18°, 10.53°, 12.97°, and 22.20° (2θ) which are completely superimposable to those shown in the simulated pattern obtained from single crystal XRD diffraction data; however, additional peaks evidence the rising of new crystal phases. To get additional insight, XRD powder diffraction were recorded during 10 min on wet crystals of [L_2_Cu_2_(CH_3_CN)_2_]•2­(CH_3_CN), acquiring a spectrum each 2 min (Figure S3).

**Figure 2:**
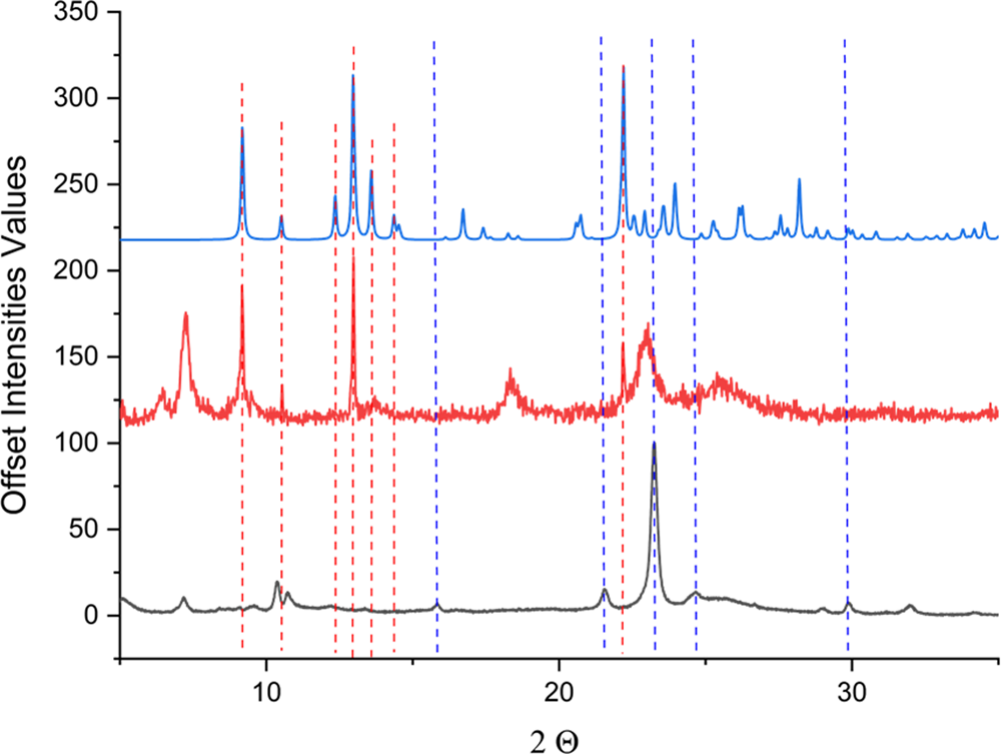
Plot of the simulated PXRD pattern (blue line), the wet crystals with acetonitrile (red line), and the dried and ground crystalline powder (black line). The red and blue dotted lines emphasize the characteristic reflections of the simulated pattern for [L_2_Cu_2_(CH_3_CN)_2_]•2CH_3_CN (red), along with the new reflection observed after drying under vacuum (blue).

All of the acquisitions clearly highlight a crystalline phase change with a loss of long-range crystallinity upon exposure to moist air (Figure S3) and the formation of a new crystalline form upon air exposure or taking to dryness under vacuum (pale blue line, Figure 2). The nature of [L_2_Cu_2_(H_2_O)_2_]•2­(H_2_O) was demonstrated by analytical, magnetic, and spectroscopic data. Elemental analysis data matches for the presence of four water molecules linked to the Cu_2_L_2_ core, while magnetic susceptibility measurements yielded a value of 2.826 μB for μeff, consistent with the presence of one unpaired electron per copper core, as expected for d^9^ ions, particularly in a dianion O,N coordination environment toward Cu­(II) cations. However, the dynamic nature of the compound in both solid and solution states was detected by EPR measurements. Electronic signals were recorded, ruling out antiferromagnetic coupling, but RT and 77K studies did not yield resolved peaks both in the solid state and in acetonitrile solutions (Figures S4 and S5).^33–36^ The temperature variations as well as the removal of oxygen in solvents did not help to solve the spectra, remaining unresolved unless a strongly coordinating pyridine was used as solvent (Figure S6). In the IR spectra, recorded in a solid sample left to moist air, the C=O stretching frequencies fall around 1680 cm^–1^ and 1660 cm^–1^, while for the free ligand L, they fall at 1760 cm^–1^ and 1720 cm^–1^; this redshift magnitude is diagnostic of carbonyl coordination to the metal ion as a monodentate ligand.^36^ In addition, the redshift of 12 cm^–1^ for the ν_C–H_ band of the secondary amide and a large redshift of 23 cm^–1^ of the ν_C=N_ band of the triazole ligand were also observed (Figure S3, Supporting Information). Additional bands can be tentatively attributed to Cu–O and Cu–OH_2_ vibrational stretching modes at 529 cm^–1^ and 538 cm^–1^,^37^ while the Cu–N vibrational stretching modes, likely falling at 256 and 248 cm^–1^, are attributed after the assignment of the rocking and wagging water librational modes at 784 cm^–1^ and 612 cm^–1^,^38^ become active after water coordination to Cu­(II) ions (Figure S7, Supporting Information). All of the aforementioned data are consistent with N,N′, and O,O′ coordination of the ligand to Cu­(II) ions and the additional coordination of water molecules (Table S2 and Figure S7). The crystal structure demonstrates the presence of two coordinated CH_3_CN and entrapped CH_3_CN molecules for each [L_2_Cu_2_] core; however, after the crystals were removed from the mother liquor, upon exposure to moist air, the displacement of the acetonitrile by water molecules is readily detected. Experimental details substantiate this behavior; in fact, the solid-state IR spectrum shows peaks due to water librational modes (Figure S8),^39^ and the elemental analysis and the weight losses recorded in the TGA profiles corroborate the presence of four molecules of water (Figures S9 and S10). In detail, the TGA decomposition curve under pyrolytic conditions (N_2_) records the first weight loss from 129 °C, highlighting the loss of four bound water molecules (129–448 °C, −52.37%) (−4H_2_O) beyond the loss of four trifluoroacetamido moieties from the ligand (−4COCF_3_), followed by a second degradation step (448–514 °C, −10.87%) due to definitive loss of substituents affording 1,2,4-triazole species (−4NH_2_),^40^ and a third weight loss (514–746 °C, −17.07%) matching the complete decomposition of the organic part, leaving a residual mass corresponding to CuO. Moreover, the square based pyramidal (SBP) geometry at the solid state was confirmed by the electronic ATR diffuse spectrum consisting of an intense green light reflection at 522 nm, beyond a high energy peak at 14492 cm^–1^ (690 nm) and a low energy peak at 11049 cm^–1^ (905 nm), with the latter attributable to electronic d-d transitions (see Figure S11).^41,42^ In acetonitrile or ethanol solutions, only a d-d transition is observed at 680 or 650 nm, respectively, as reported in Figure 3 and Figures S12 and S13 in Supporting Information. ^1^H, ^19^F and ^13^C NMR characterizations were not helpful to characterize the behavior in solution both for the paramagnetism of the compound, and for the likely dynamic acetonitrile/water and solvent exchanges (Figures S14–S16). Conversely, the tendency to lose water or acetonitrile molecules and to aggregate in solution were detected by (−) ESI-MS data in acetonitrile and methanol, showing fragments at *m*/*z* 702.6 and 1408.2, corresponding to [L_2_Cu_2_–H]^−^ monomeric and [L_2_Cu_2_–H]_2_^–^ dimeric species, respectively (see Figures S17 and S18 in Supporting Information). To get additional insight into the nature of the compound in solution and verify its tendency to aggregate, UV–visible spectra were acquired in ethanol (Figure 3) and acetonitrile (Figures S12, S13 and S19, Supporting Information) solvents. Data obtained from the UV–vis dilution experiments were analyzed by using the Cooperative Equilibrium Kinetics (CoEK) model (Figure S20). The CoEK model postulates that the initial association event is distinct from subsequent ones (*K*_d_ ≠ Ke), and the fit of the dilution data yields an error of roughly 1%. These findings suggest that self-association is likely to occur through a positively cooperative mechanism, with the initial dimer facilitating the aggregation of additional monomers, resulting in the formation of a linear supramolecular polymer (Ke > *K*_d_; see Table S3 and Scheme S2 in the Supporting Information).^43,44^ The experimentally determined equilibrium constants (Ke) for the complex (Ke = 10^3^–10^2^ M^–1^, see Table S3) significantly exceed those experimentally obtained for the self-assembly of small anionic compounds, for example, sulfonate (Ke = 100 M^–1^) or carboxylate (Ke = 10^1^ M^–1^).^45,46^ The polymerization of [L_2_Cu_2_(solv)_2_]•2­(solv) is likely enhanced by the presence of Cu­(II)–Cu­(II) metallophilic interactions, as similarly reported for Cu­(II) complexes with substituted aroylhydrazones and azo-dyes.^40,46^ Moreover, the polymer formation is underscored by the moderate blue-shift observed in the UV–vis absorption metal-centered bands at 680 nm (Δλ = 10 nm, Ethanol 2.56 mM solution) and 650 nm (Δλ = 10 nm, CH_3_CN 2.56 mM solution) upon dilution; the shifts were attributed to the effect of the dilution on the self-assembling (see insect image in Figure 3).^46^ The herein calculated *K*_d_ is plausible as it is of the same order of magnitude as those reported for Cu­(II) ions and N-donor chelating ligands (10^2^ to 10^3^ M^–1^), recently documented by Jehdaramarn et al.^47^

**Figure 3:**
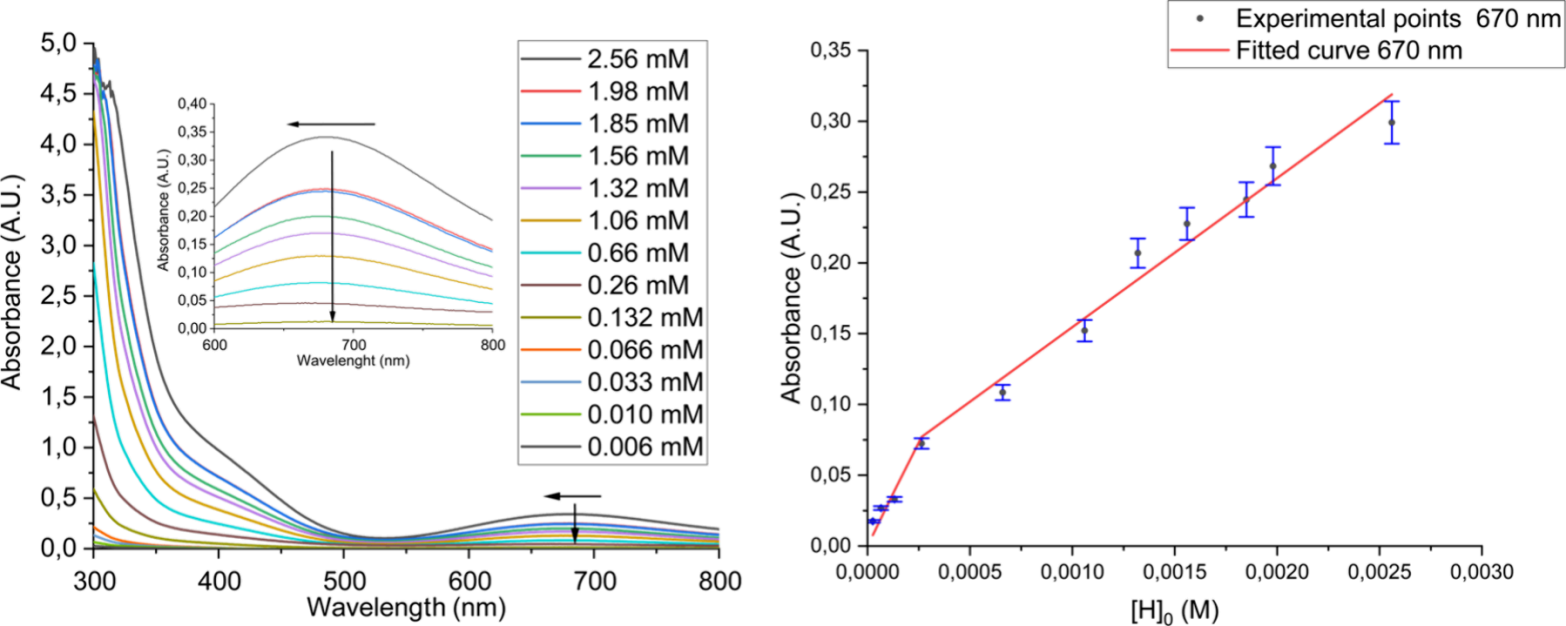
UV–vis spectroscopy dilution experiment in an ethanol solution. Inset: enlargement of the 600–800 nm region (left). CoEK model data fitting for UV–vis dilution experiments in ethanolic solutions reporting error bars within 5% (right).

Given the ready displacement of solvent molecules from the L_2_Cu_2_ core leaving the copper center mostly available, [L_2_Cu_2_(solv)_2_]•2­(solv) dissolved in CH_3_OH or CH_3_CN was evaluated as a catalyst for the homocoupling of phenylacetylene to obtain 1,4-diphenylbutadiyne. The results and experimental conditions are shown in Table 1. The best conversion to the homocoupled product is obtained at room temperature with 24 h of reaction time using DBU as the base in 20% molar loading and 2.5 mol loading of catalyst (yield 71%, see Table 1, entry 7), avoiding the formation of acetophenone that was detected by GC-MS analysis when using water as base (entry 1, Table 1, 4%, see Figure S21 of the Supporting Information). This subproduct is formed due to the side addition reaction of water to phenylacetylene.^48^ By reducing the catalyst and base loadings, suboptimal outcomes were obtained, with rather satisfactory yields with 1% mol of catalyst and 50% mol of DBU (Table 1, entry 5). Notably, the catalyst [L_2_Cu_2_(H_2_O)_2_]•2­(H_2_O) demonstrates better performance compared to CuCl_2_ (10% mol), which yielded only 15% of conversion after 6 h at room temperature (Table 1 entry 8). Overall, the catalyst exhibited satisfactory performance, particularly considering the straightforward nature of the experimental procedures and conditions, like ambient temperature conditions, without the necessity for palladium as a cocatalyst.^49^ Additionally, the purification techniques used were minimal, requiring only filtration through a silica pad and evaporation under reduced pressure, as highlighted by the ^1^H NMR spectrum reported in Figure 22S.

**Table 1:** Reaction Conditions for the Phenylacetylene Homocoupling Reaction Catalyzed by [L_2_Cu_2_(solv)_2_]•2­(solv)

Entry	Catalyst (% mol)	Base (% mol)	Solvent	Conditions^a^	Yield (%)	TON
1	2.5	H_2_O, excess	CH_3_OH	24 h, 110 °C	30 + 4%^b^ ^c^	12
2	1	DBU, 20	CH_3_CN	3 h, R.T.	25^d^	25
3	1	DBU, 20	CH_3_CN	6 h, R.T.	40^d^	40
4	1	DBU, 20	CH_3_CN	24 h, R.T.	50^d^	50
5	1	DBU, 50	CH_3_CN	24 h, R.T.	62^d^	62
6	2.5	DBU, 20	CH_3_CN	24 h, R.T.	64^d^	25.6
7	2.5	DBU, 50	CH_3_CN	24 h, R.T.	71^d^	28.4
8	CuCl_2_, 10	DBU, 20	CH_3_CN	6 h, R.T.	15^e^	1.5

a A typical catalytic reaction was conducted by dissolving 1 mmol of phenylacetylene in 2 mL of solvent; the reaction was monitored by TLC (eluent = 95/5, petroleum ether/ethyl acetate). Phenylacetylene rf = 0.85; 1,4-diphenylbutadiyne, 1, rf = 0.7; acetophenone rf = 0.40.

b The presence of H_2_O yields acetophenone as a byproduct.

c The conversion of phenylacetylene to the products was estimated by using GC-MS.

d Yields were calculated after elution on a silica gel pad (eluent = 95/5, petroleum ether/ethyl acetate).

e Refer to ref 50.

In conclusion, a dinuclear square pyramidal copper­(II) complex with a 3,5-bis­(trifluoromethylacetamido)­triazolyl-2yl (L) N,O,N′,O′ chelating ligand was prepared. The characterization of the complex highlights the formation of a neutral dinuclear moiety of the type N,N′,O,O′ Cu_2_L_2_, exhibiting dimeric square pyramidal geometry with the apical coordination of an acetonitrile molecule per copper­(II) center; moreover, two additional molecules of acetonitrile were found entrapped in the structure voids. Of note, the title compound displays an atypical easy displacement of the acetonitrile molecules by water molecules even in the solid state. By considering this property, this complex was ascertained in phenylacetylene homocoupling reactions; the results, if compared to other catalysts (Table S4, Supporting Information), label the compound as a rather good homogeneous catalyst, whose performance is featured by low catalyst loadings (1–2.5% mol) and up to 60% of conversion in the presence of DBU as the base. The solvent effect on the catalyst reactions was explained by analyzing the cooperative self-aggregation of the complex in acetonitrile and ethanol solutions.

## Supplementary Material



## Data Availability

The data underlying this study are available in the published article and its Supporting Information.

## Notes

Experimental details, table of Crystallographic Parameters, crystal packing figures, IR and ^1^H, ^19^F NMR spectra, TGA and ESI-MS plots, UV–visible spectra and dilution experiments data, catalytic GC results (PDF)
