# Supplementary material for: An Atypical Dinuclear Copper(II) 1,2,4-Triazolyl Complex as a Catalyst for Palladium-Free Csp-Csp Homocoupling of Phenylacetylene
Source: ACS Org Inorg Au. 2026 Apr 14;6(3):270–6. doi: 10.1021/acsorginorgau.6c00013 (PMC13237604; doi:10.1021/acsorginorgau.6c00013)
Supplement: Supplementary file 1 [file gg6c00013_si_001.pdf]

Supporting Information

for

An Atypical Dinuclear Copper(II) 1,2,4-Triazolyl Complex as a Catalyst for Palladium-free Csp-Csp Homocoupling of Phenylacetylene

Lorenzo Luciani,[1] Nicola Sargentoni, [1] Magda Monari[2] and Rossana Galassi,[1]*

1 *University of Camerino, School of Science and Technology, Chemistry Division, CHIP, Via Madonna delle Carceri, Camerino*

2 *University of Bologna, Chemistry Department, "Giacomo Ciamician", Via Gobetti 85, Bologna*

* Corresponding author, email: rossana.galassi@unicam.it

Table of contents

Crystallography page 3

Table S1. Crystallographic data page 3

Figure S1, view molecular structure page 4

Figure S2, molecular unit packing page 5

Figure S3, XRD powder pattern over time page 5

Synthesis and main characterizations page 6

Synthesis of the ligand L page 7

Synthesis of the $[L_2Cu_2(solv)_2]$•2(solv) page 7

Figure S4 EPR spectra for solid $[L_2Cu_2(solv)_2]$•2(solv) page 8

Figure S5 EPR spectra for $[L_2Cu_2(solv)_2]$•2(solv) page 8

Figure S6 EPR spectra for $[L_2Cu_2(solv)_2]$•2(solv) in pyridine page 9

Table S2, FT IR data page 9

Figure S7, IR spectra page 10

Figure S8, IR spectra page 11

Figure S9, TGA Plots page 11

Figure S10, TGA Plots page 12

Figure S11, ATR spectrum of solid $[L_2Cu_2(solv)_2]$•2(solv) Page 12

Figure S12, UV- visible spectrum of $[L_2Cu_2(solv)_2]$•2(solv) in page 13

Figure S13, UV- visible spectrum of $[L_2Cu_2(solv)_2]$•2(solv) in page 13

Figure S14, ^{1}H and ^{19}F NMR spectra of the Ligand L page 14

Figure S15, ^{1}H and ^{19}F NMR spectra of the L and $[L_2Cu_2(solv)_2]$•2(solv) page 15

Figure S16, ^{13}C NMR spectra of the L and $[L_2Cu_2(solv)_2]$•2(solv) page 16

Figure S17, ESI-MS m/z spectrum $[L_2Cu_2(solv)_2]$•2(solv) in Ethanol page 17

Figure S18, ESI-MS m/z spectrum $[L_2Cu_2(solv)_2]$•2(solv) in CH_3CN page 18

UV-visible spectroscopy experiments page 19

Table S3. Calculated Kd in acetonitrile page 19

Figure S19, UV-visible dilution experiments page 20

Figure S20, data fitting page 20

Scheme S2, self-association equilibria page 21

Catalysis page 22

Figure S21, GC plots page 22

Figure S22, ^{1}H NMR 1,4-diphenylbutadiyne $CDCl_3$ page 23

Table S4. Phenylacetylene homocoupling reactions data page 24

References page 25

Crystallography

The X-ray intensity data for **[$L_2Cu_2(CH_3CN)_2$]•2CH_3CN** were collected on a Bruker APEX-II CCD diffractometer using Mo−Kα radiation. All data were processed using the Bruker suite of programs [1−3], and the structure was solved by direct methods and refined with the SHELX program suite [4, 5] in the space group *$P2_1/c$*. All non-hydrogen atoms were assigned anisotropic displacement parameters. The hydrogen atoms were placed in idealized positions and included as riding with constrained isotropic displacement parameters (C—H = 0.98 Å for methyl protons and N—H = 0.86 Å for amide protons and refined as riding with $U_{iso}(H)$ = 1.5$U_{eq}(C)$ or 1.2$U_{eq}(N)$). The asymmetric unit contains, in addition to one half of **[$L_2Cu_2(CH_3CN)_2$]**, one crystallization CH_3CN molecule. The Cu complex lies around an inversion centre to which all the atoms conform except the amidic hydrogens that are disordered over two symmetry equivalent positions with occupation factors of 50%. One of the CF_3 groups is disordered, and the fluorine atoms are split over two positions with occupation factors of 75 and 25%, respectively. Molecular graphics were generated using the program Mercury [6]. **Table S1** reports crystal data and refinement parameters for **[$L_2Cu_2(CH_3CN)_2$]•2CH_3CN.**

The quick PXRD acquisition on crystals deposited with a drop of acetonitrile mother liquor was acquired in the 2θ range 5.0-35.0°, with steps of 0.02° and a time per step of 0.05 s (total time = 1 minutes and 25 s) with a Bruker D6 Phaser diffractometer, equipped with Ni-filtered Cu Kα radiation (λ = 1.5418 Å), a Lynxeye linear position-sensitive detector, and the following optics: primary-beam Soller slits (2.3°), fixed divergence slit (0.5°), receiving slit (8 mm). The generator was set at 40 kV and 15 mA.

The PXRD patterns of the ground and dried crystalline powder were acquired in the 2θ range 5.0-35.0°, with steps of 0.01° and a time per step of 2 s (total time = 1 hour and 50 minutes) with a Bruker D6 Phaser diffractometer, equipped with Ni-filtered Cu Kα radiation (λ = 1.5418 Å), a Lynxeye linear position-sensitive detector, and the following optics: primary-beam Soller slits (2.3°), fixed divergence slit (0.5°), receiving slit (8 mm). The generator was set at 40 kV and 15 mA.

For the sake of comparison, the acquired PXRD patterns were normalized in the 0-100 range of intensities, and the baselines were recalculated by using Origin Pro software.

Table S1. Crystal data and structure refinement for **[$L_2Cu_2(CH_3CN)_2$]•2CH_3CN**.

Compound	**[$L_2Cu_2(CH_3CN)_2$]•2CH_3CN**
Formula	$C_{16}H_8Cu_2F_{12}N_{12}O_4$ •2CH_3CN
Fw	869.53
T, K	296
λ, Å	0.71073
Crystal symmetry	Monoclinic
Space group	*$P2_1/c$*
a, Å	8.4347(17)
b, Å	13.647(3)
c, Å	13.605(3)
α	90
β	94.481(5)
γ	90
Cell volume, $Å^3$	1561.3(6)
Z	2
D_C, Mg m^{-3}	1.850
Absorption coefficient, mm^{-1}	1.489
F(000)	860
Crystal size/ mm	0.30 x 0.25 x 0.25

θ limits, °	2.985 to 25.495
Reflections collected	8584
Unique obs. Reflections [$F_o > 4\sigma(F_o)$]	2824 [R_{int} = 0.0942]
Goodness-of-fit-on F^2	1.070
R_1 (F)[a], wR_2 (F^2) [I > 2σ(I)]	0.0691, 0.1825
Largest diff. peak and hole, e. Å^{-3}	1.013 and -1.273
CCDC	2396411

a) $R_1 = \Sigma||F_o|-|F_c||/\Sigma|F_o|$.[b] $wR_2 = [\Sigma w(F_o^2-F_c^2)^2/\Sigma w(F_o^2)^2]^{1/2}$ where $w = 1/[\sigma^2(F_o^2) + (aP)^2 + bP]$ where $P = (F_o^2 + F_c^2)/3$.

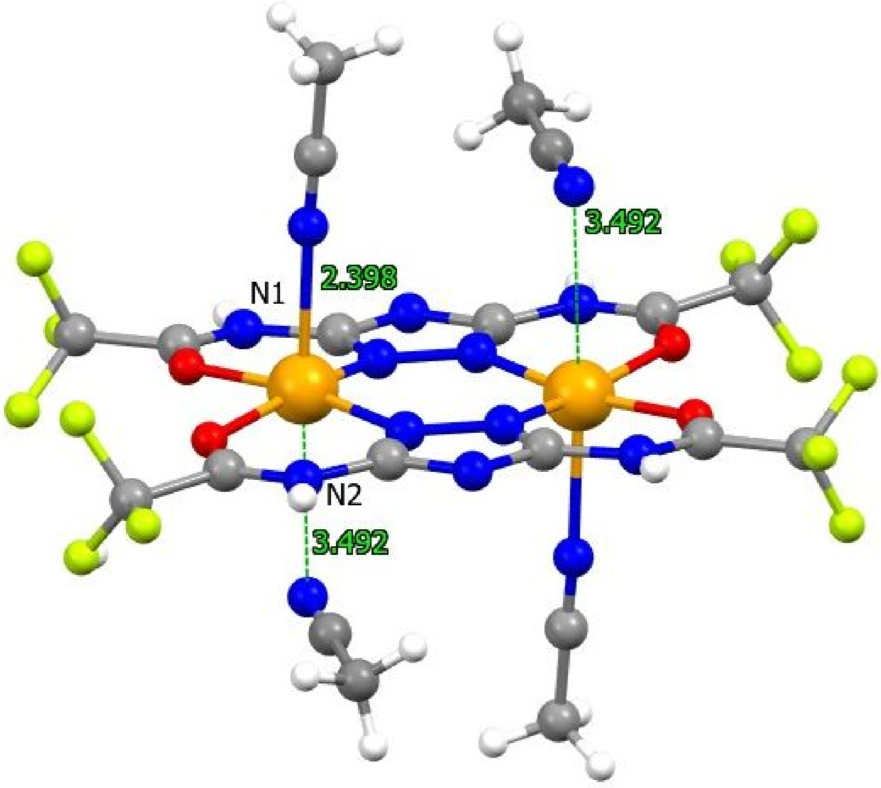

Figure S1. Molecular structure showing the coordinated and the non-coordinated CH_3CN molecules (green dotted lines) in $[L_2Cu_2(CH_3CN)_2]$•$2(CH_3CN)$.

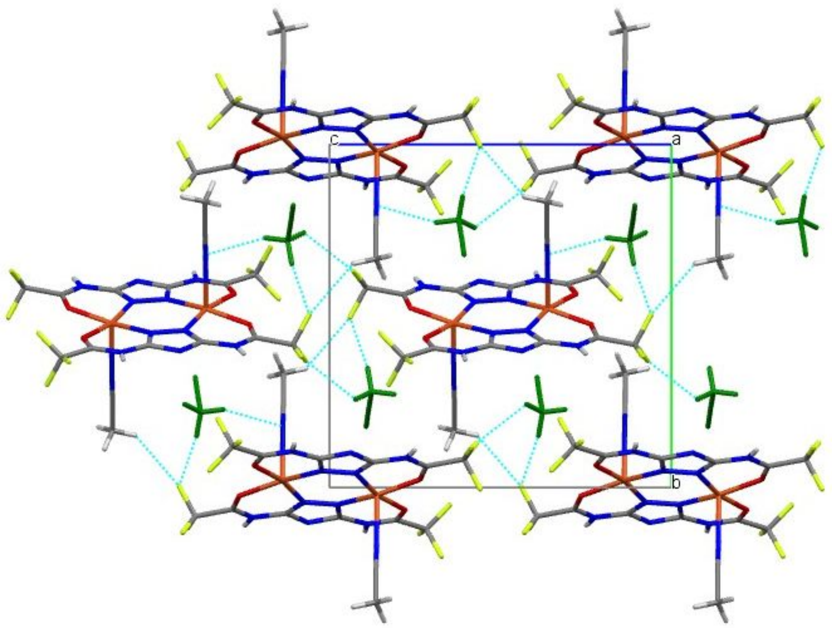

Figure S2. View down the *a* axis of the crystal packing of [$L_2Cu_2(CH_3CN)_2$]•2(CH_3CN). C-H···F H bondings (light blue dotted lines) involving the coordinated and ~~the~~ non-coordinated CH_3CN molecules are shown.

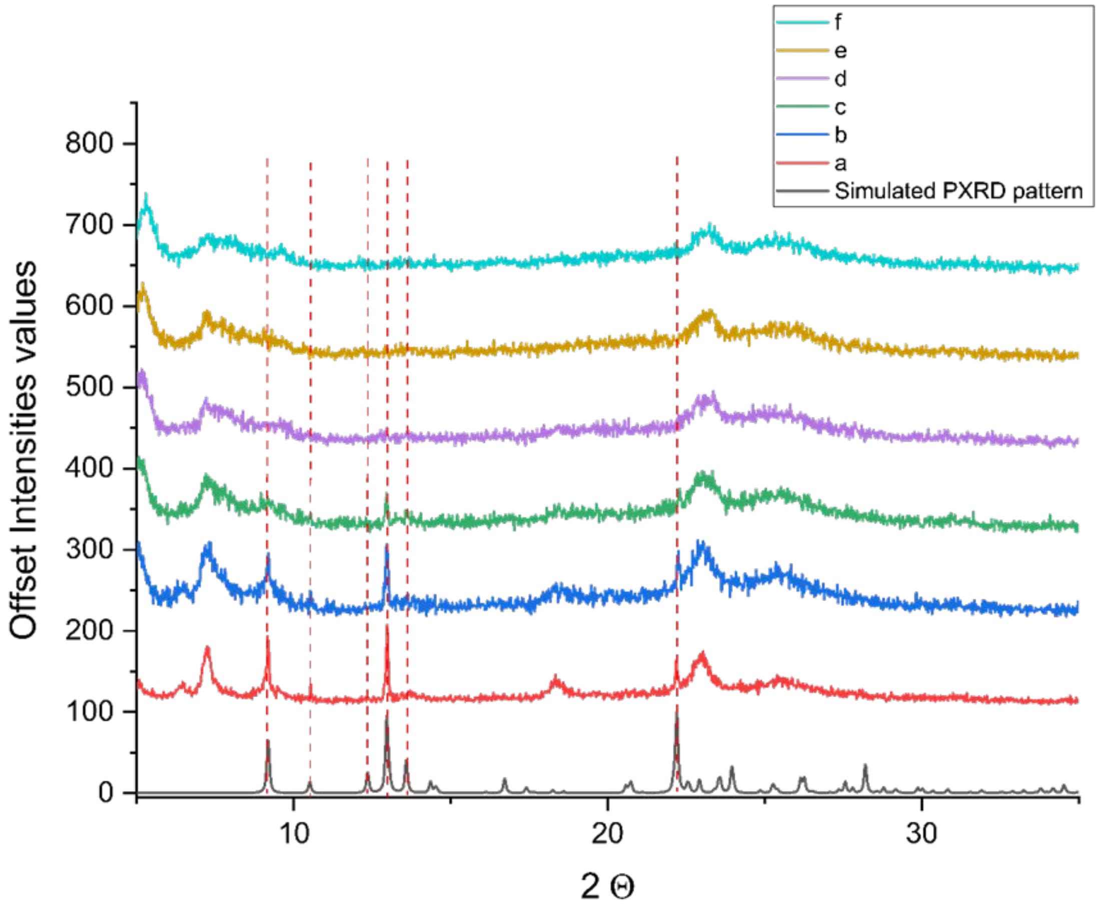

Figure S3. Plot of simulated (bottom, black spectrum) and experimental XRD powder diffractograms by exposing wet [$L_2Cu_2(CH_3CN)_2$]•2(CH_3CN) crystals to moist air for 0 a), 2 b), 4 c), 6 d), 8 e) and 10 minutes f).

Synthesis

Materials: All the reactants needed for the syntheses were purchased from Merck and used without further purification. Solvents were bought from Carlo Erba (Milano, Italy) and freshly distilled before use. Celite and molecular sieves were bought from Supelco (Merck).

Spectroscopic and analytical characterization: Elemental analyses (C, H, N, S) were performed in-house with a Fisons Instruments 1108 CHNS-O Elemental Analyser. FT-IR spectra were recorded from 4000 to 100 cm^{-1} (Mid-IR and Far-IR ranges) with a Perkin-Elmer SPEC- TRUM ONE System FT-IR instrument. IR annotations used: m = medium, s = strong, sh = shoulder, vs = very strong, and w = weak. ^{19}F NMR spectra were recorded on a 500 Bruker Ascend (500 MHz for ^{1}H). Chemical shifts, in ppm, for ^{19}F NMR spectra are relative to internal $CFCl_3$. NMR annotations, s = singlet, br = broad. Thermogravimetric analysis (TGA) was performed under a N_2 flow (10 mL min) with a PerkinElmer Simultaneous Thermal Analyzer STA 600, by setting a heating ramp from 30.00 °Cto 750.00 °C (10 °C min^{-1}). UV-Vis spectra were acquired using the Shimadzu UV-2700i spectrophotometer, equipped with the Shimadzu CPS-100 Peltier, at 298 K (Shimadzu, Kyoto, Japan). Electrospray ionization mass spectra (ESI-MS) were obtained in positive- or negative-ion mode on high-performance liquid chromatography (HPLC) Alliance 2695 Waters coupled with a single quadrupole mass spectrometry (Waters Micromass ZQ, Milford, MA, USA). The mobile phase was acetonitrile or ethanol, and the compounds were dissolved in the mobile phase with an approximate concentration of 0.1 mM. The injection volume was 1 μL, and the flow rate was 200 μL min^{-1}. Nitrogen was employed both as a drying and a nebulizing gas. Capillary voltages were typically 4000 V and 3500 V for the positive- and negative-ion modes, respectively. Confirmation of all major species in the ESI-MS study was aided by comparing the observed and predicted isotope distribution patterns, the latter calculated using the software ChemDraw Ultra 12. The GC-MS experiments were conducted using an Agilent 8890 Gas Chromatographer equipped with an autosampler Agilent 7650, a column HP-5 30 m (250-0.25 μm), and a Mass Spectrometer Agilent 5977B. Thermal desorption was carried out at 280 °C by injecting 1 μL of sample in a split mode 5:1. The gas carrier was helium injected at a constant flow rate of 1 mL/min. The oven temperature was set at 45°C and was increased to 325°C at 25°C/min, with a total run time of 12.2 minutes. The Mass spectrometer was set in SCAN mode from 29 to 500 amu. The oxidation state of copper centers was calculated by measuring magnetic susceptibility. The magnetic susceptibility was measured at 293 K by the Gouy method with a Sherwood Scientific magnetic balance MSB-Auto, using $HgCo(NCS)_4$ as a calibrant, and they were corrected for diamagnetism with the appropriate Pascal constants. The magnetic moments (in Bohr Magnetons, B.M.) were calculated by using the following equation [7]:

$$\mu_e ff = 2.828 \cdot \left(\chi_m^{corr}\, T\right)^{1/2}$$

χ_m^{corr} = corrected magnetic susceptibility

T = temperature in K

Solid-state UV-Vis-NIR reflectance spectrum was acquired using a PerkinElmer Lambda 35 spectrophotometer equipped with a lab-sphere RSA-PE-20 reflectance spectroscopy accessory. Blank was acquired using LabSphere-certified reflectance standards and was subtracted from the sample signals. The total reflectance spectrum was elaborated by calculating the baseline, smoothing with a Savitzky-Golay algorithm, and normalization to the 0-1 range.

X-band EPR measurements have been carried out on a Bruker EMX/Xenon spectrometer system equipped with a microwave frequency counter and a Nuclear Magnetic Resonance (NMR) Gauss meter for field calibration. For data acquisition in the solid state, the instrument parameters were set up as follows: Modulation Frequency: 100 kHz; Time Constant: 0.16 ms; Sweep Width: 1800 G; Modulation Power: 10.54 mW; Modulation Amplitude: 3.0 G; Sweep Time: 120 s; in solution some instrument parameters were changed as follows: Sweep Width: 600 G; Modulation Amplitude: 1.0 G; Sweep Time: 240 s.

Synthesis of ligand L. L was prepared by a slightly modified procedure reported by Selassie *et al.* [8]. To a suspension of 3,5-diamino-1,2,4-triazole (0.5 g, 5.045 mmol) in 20 mL of dry toluene, trifluoromethanesulfonic anhydride (2.1 g; 10.1 mmol) was added; the suspension was stirred at reflux for 7 h under a nitrogen atmosphere. The white microcrystalline precipitate was subsequently collected by filtration, washed with diethyl ether (5 x 15 mL), and dried at reduced pressure. Yield 1.110 g, 76%.

^{19}F-NMR (CD_3CN, 500 MHz, 293K) δ: -76.16 (s).

^{1}H-NMR (CD_3CN, 500 MHz, 293K) δ: 10.85 (s, br).

^{13}C NMR (CD_3CN, 500 MHz, 293K) δ: 155.53 (s), 155.21 (s), 149.11 (s, br), 116.59 (s), 114.32 (s).

IR (cm^{-1}): 3358 (w, sh), 3265 (w), 3202 (w), 3087 (w), 3053 (w), 2880 (w), 1760 (m), 1722 (m), 1698 (m, sh), 1652 (m), 1621 (m), 1564 (s), 1558 (s), 1520 (m), 1433 (w), 1377 (m), 1318 (m), 1276 (m), 1169 (vs), 1160 (vs), 1070 (s), 1022 (m), 927 (m), 892 (w), 815 (m), 759 (m), 720 (s), 694 (m, sh), 681 (w), 648 (w), 600 (w), 577 (w), 518 (m), 491 (m), 479 (m), 434 (m), 427 (m), 399 (w, sh), 354 (w), 349 (w), 327 (m), 301 (w), 290 (w), 280 (w), 270 (w), 242 (m, sh), 231 (s), 202 (w), 185 (w), 175 (m), 169 (m), 159 (m), 149 (m), 140 (s), 131 (m), 121 (s).

Anal calcd for $C_6H_3F_6N_5O_2$: C, 24.75; H, 1.04; N, 24.06. Found: C 24.71; H 1.00; N 24.11.

LRMS (ESI) m/z: $[L - H]^-$, calculated for $C_6H_2F_6N_5O_2$ 290.01, found 290.0.

Synthesis of complex $[L_2Cu_2(solv)_2]\cdot 2(solv)$

To a solution of the ligand L in 25 mL of CH_3CN (200 mg; 0.68 mmol), Cu_2O (58 mg; 0.44 mmol) was added. The suspension turned green after two hours and was stirred overnight. The suspension was filtered on a celite bed, and the solution was dried at reduced pressure. The green powder was washed with diethyl ether (2 × 10 mL) and dried at reduced pressure. XRD-quality green crystals of $[L_2Cu_2(CH_3CN)_2]\cdot 2(CH_3CN)$ were obtained by slow evaporation of a CH_3CN solution at room temperature over 48 hours. Yield 0.180 g ,70%. The exposure of the crystals to moist air or taking to dryness the crystals result in the formation of a green microcrystalline powder of $[L_2Cu_2(H_2O)_2]\cdot 2(H_2O)$ as evidenced by the following characterization.

^{19}F-NMR (CD_3CN, 500 MHz, 293K) δ: -76.31 (s, br, FWHM* = 486 Hz).

^{1}H-NMR (CD_3CN, 500 MHz, 293K) δ: 7.62 (s, br).

^{13}C NMR (CD_3CN, 500 MHz, 293K) δ: no signals were detected, even after overnight accumulations.

* Full Width Half Maximum

IR (cm^{-1}): 3441 (w), 3265 (w), 3356 (w), 3175 (w), 3060 (w), 2997 (w), 2905 (m), 2840 (w), 2747 (w), 1681 (w, sh), 1642 (w, sh), 1599 (s), 1561 (m), 1538 (s, sh), 1493 (m, sh), 1458 (w), 1416 (m), 1299 (m), 1194 (s), 1147 (s), 1111 (m, sh), 1070 (m), 932 (m), 911 (m, sh), 845 (w), 785 (m), 740 (m), 725 (m, sh), 692 (w), 614 (w), 582 (w), 538 (s), 529 (m, sh), 516 (m), 493 (m), 452 (w), 390 (w), 362 (m), 346 (w), 340 (w), 321 (s), 297 (w), 286 (w), 257 (s), 248 (s), 230 (s), 205 (w), 193 (w), 186 (w), 173 (m), 151 (s), 144 (m, sh), 136 (w), 121 (w), 115 (w), 108 (w).

Anal calcd for $C_{12}H_{10}Cu_2F_{12}N_{10}O_8$: C, 18.54; H, 1.30; N, 18.02. Found: C, 18.67; H 1.28, N 18.39.

LRMS (ESI) m/z: $[L_2Cu_2 – H]^-$, calculated for $C_{12}HCu_2F_{12}N_{10}O_4$ 702.86, found 702.8; $[L_3Cu_2 + H_2O + EtOH-H]^-$, calculated for $C_{20}H_{10}Cu_2F_{18}N_{15}O_8$ 1057.91, found 1058.5; $[L_2Cu_2 - H]_2^-$, calculated for $C_{24}H_3Cu_4F_{24}N_{20}O_8$ 1408.76, found 1408.7.

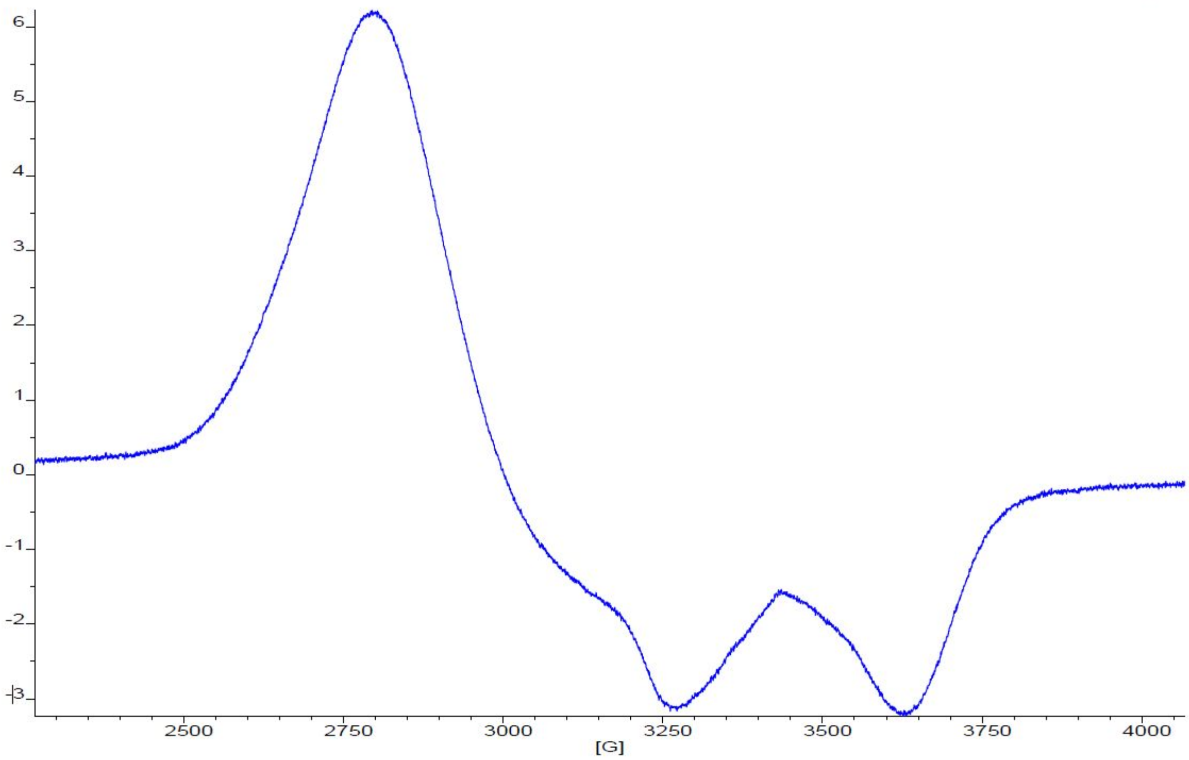

Figure S4. X band recorded at room temperature on crystals of $[L_2Cu_2(H_2O)_2] \cdot 2H_2O$**.**

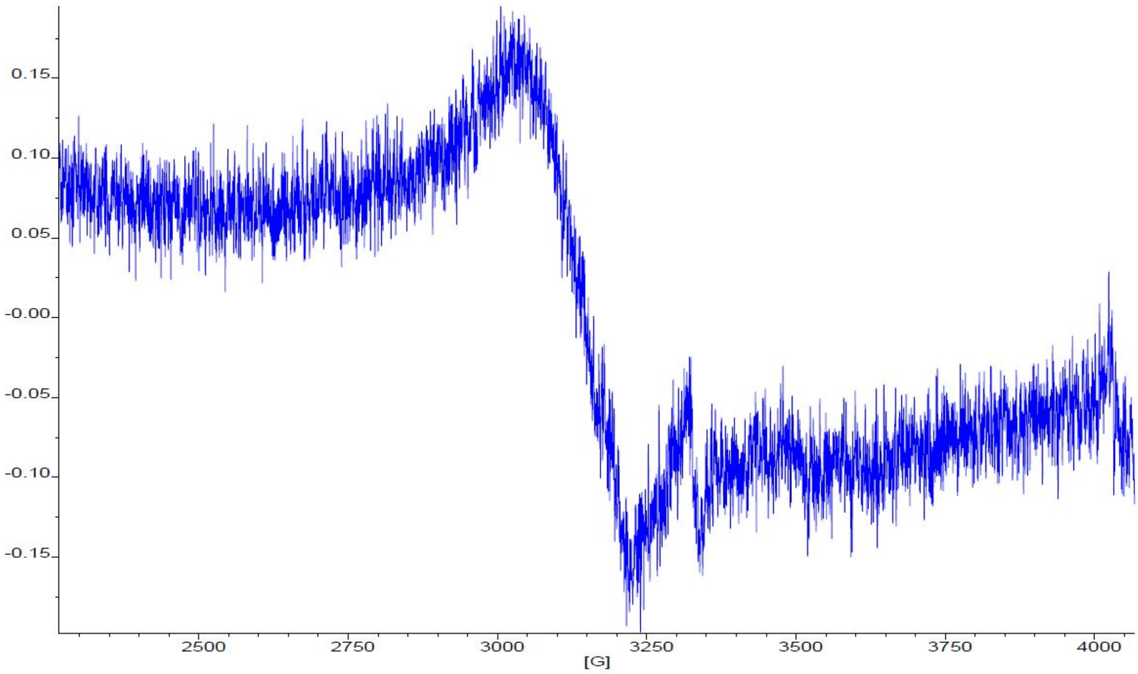

Figure S5. X band recorded at room temperature on crystals of $[L_2Cu_2(H_2O)_2] \cdot 2H_2O$ dissolved in deoxygenated CH_3CN (under argon)**.** The use of oxygenated acetonitrile did not affected the spectrum.

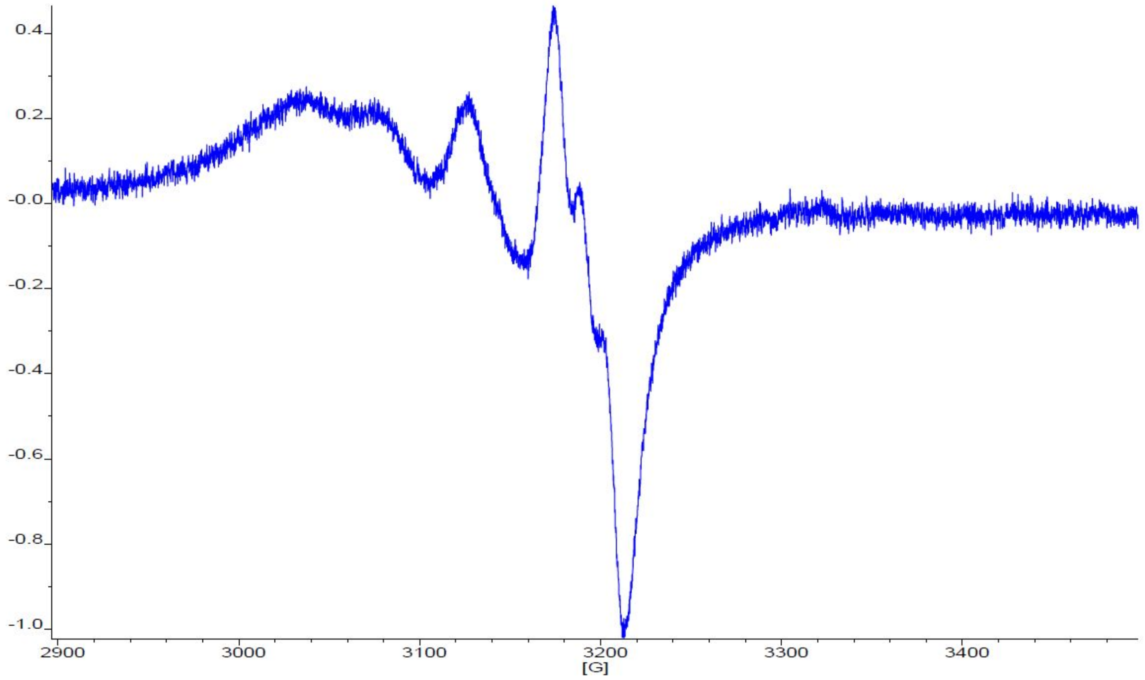

Figure S6. X band recorded at room temperature on crystals of $[L_2Cu_2(H_2O)_2]$ $2H_2O$ dissolved in deoxygenated pyridine**.** The use of oxygenated pyridine did not affect the spectrum.

Table S2. Representative FT-IR bands comparison between L and $[L_2Cu_2(H_2O)_2]$•$2(H_2O)$.

**L (cm^{-1})**	**$[L_2Cu_2(H_2O)_2]$•$2(H_2O)$ (cm^{-1})**	**Assignments**	**Δν * (cm^{-1})**
-	3441; 3200-3400 (br)	ν(O-H), H_2O	-
1760; 1720	1680; 1660	νC=O secondary amide	-80; -60
1652	1640	νN-H secondary amide	-12
1622	1599	νC=N triazole ring	-23
-	784	H_2O rocking	
	612	H_2O wagging	-
	529, 538	Cu-O bond stretching	-
	256	Cu-N stretching mode	-

* Variation in cm^{-1} of the vibrational mode absorption between the complex and the free ligand L.

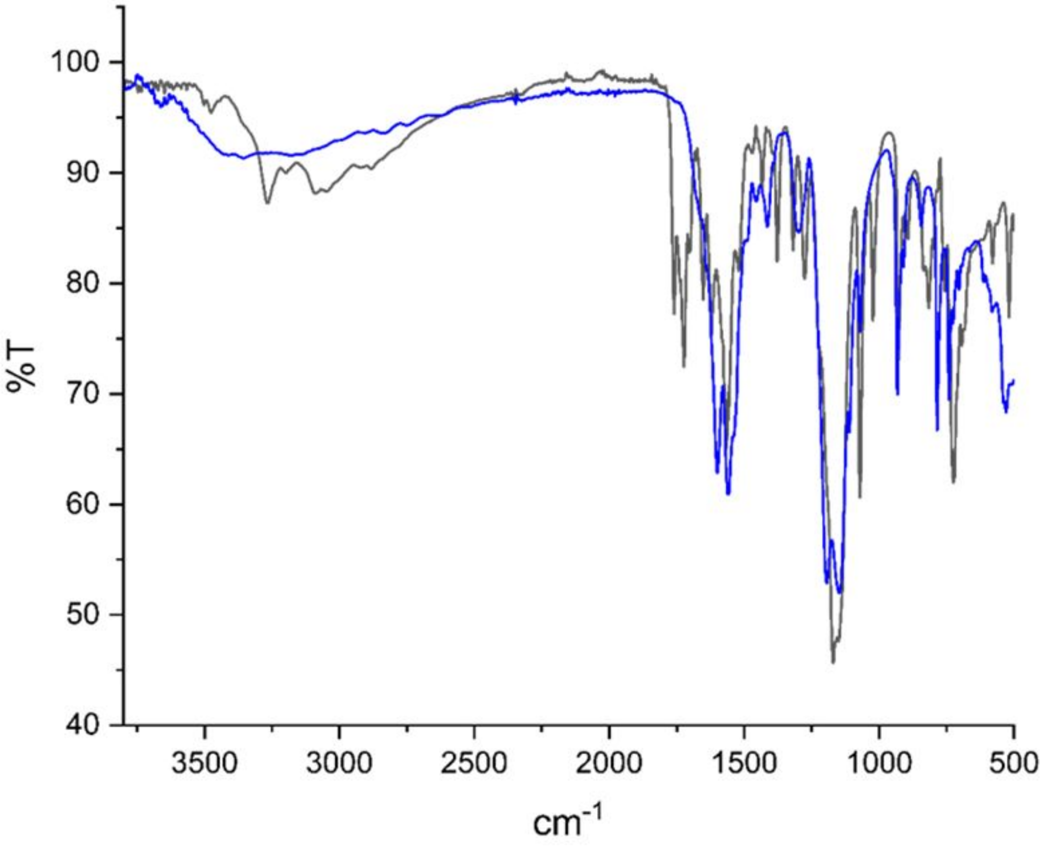

Figure S7. FT-IR in the range 4000-400 cm^{-1} of $[L_2Cu_2(H_2O)_2]•2(H_2O)$ (top) and overlapped IR spectra (bottom) of $[L_2Cu_2(H_2O)_2]•2(H_2O)$ (blue line) and L (black line).

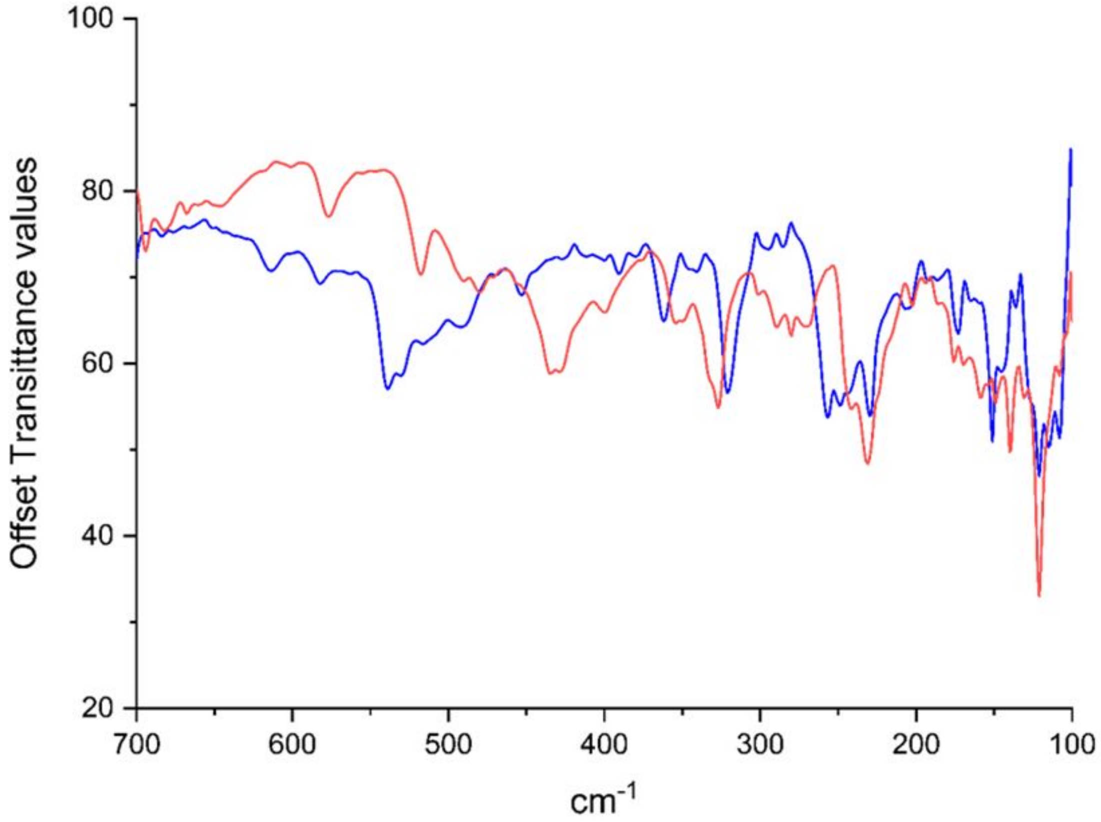

Figure S8. FT-IR in the range 700-100 cm^{-1} of $[L_2Cu_2(H_2O)_2]{\bullet}2(H_2O)$ (top) and overlapped IR spectra (bottom) of $[L_2Cu_2(H_2O)_2]{\bullet}2(H_2O)$ (blue line) and L (black line).

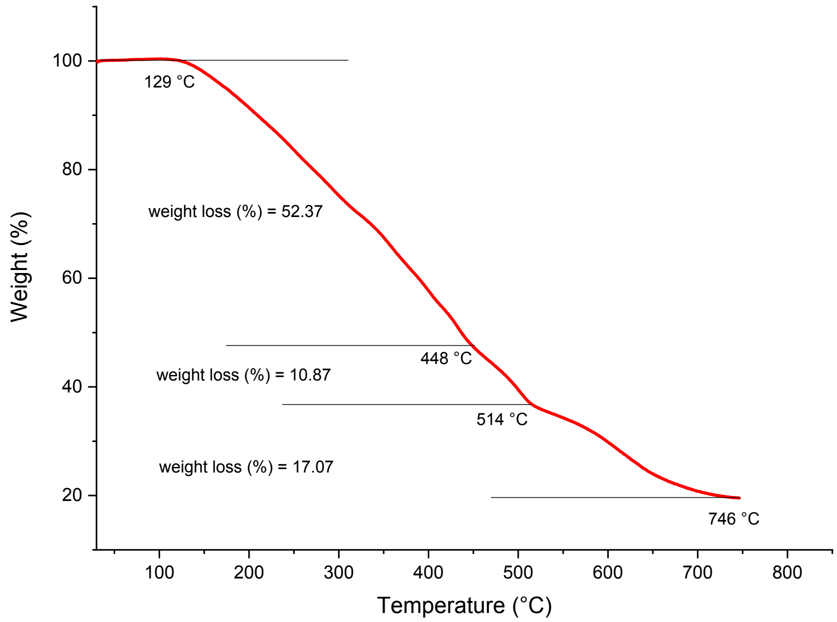

Figure S9. TGA plot of $[L_2Cu_2(H_2O)_2]{\bullet}2(H_2O)$ displaying the weight losses upon heating from room temperature to 750°C, under N_2 atmosphere.

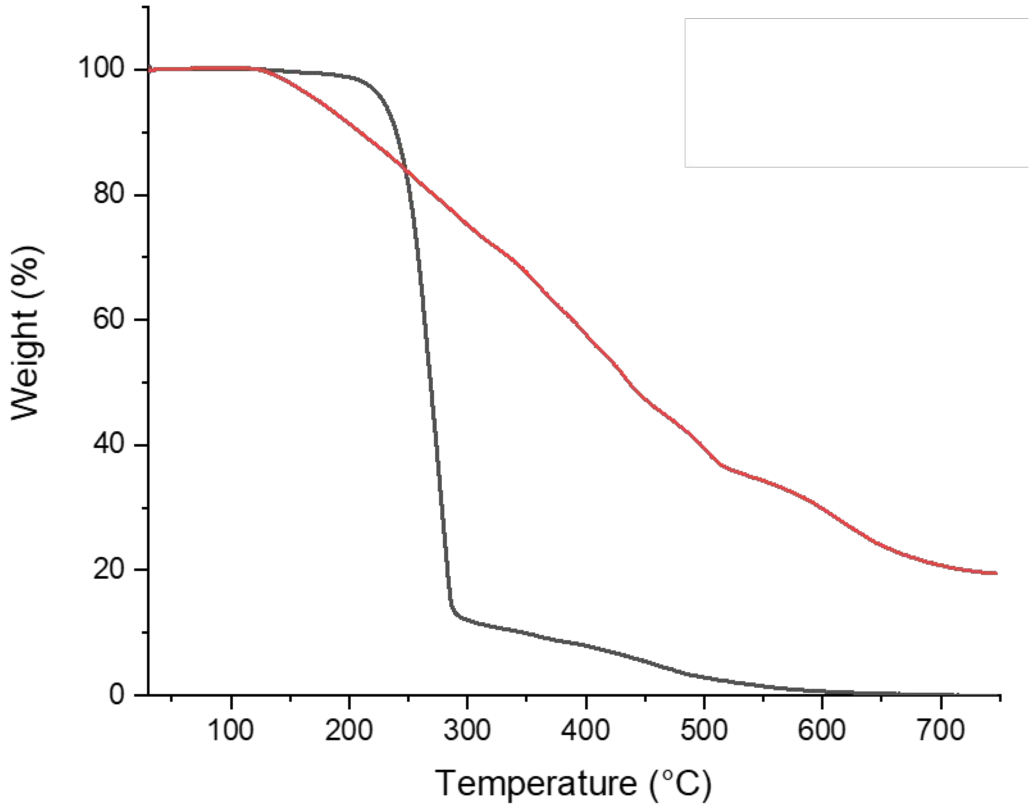

Figure S10. Overlapped TGA plots of L (black line) and $[L_2Cu_2(H_2O)_2]•2(H_2O)$ (red line) from 30°C to 750°C, under N_2 atmosphere.

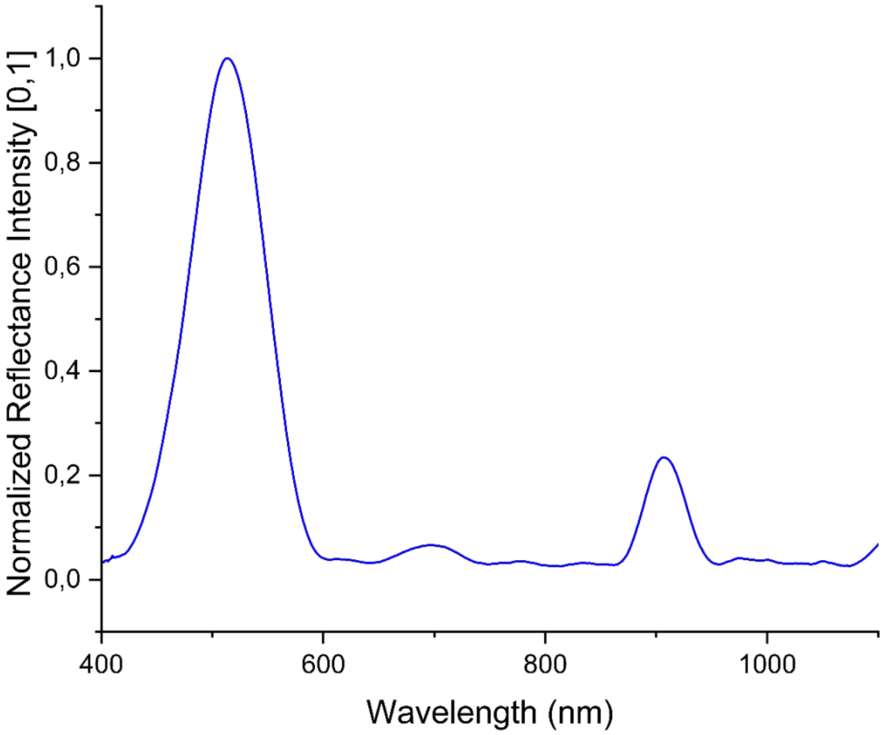

Figure S11. Normalised ATR diffuse spectrum recorded on microcrystalline $[L_2Cu_2(H_2O)_2]•2(H_2O)$.

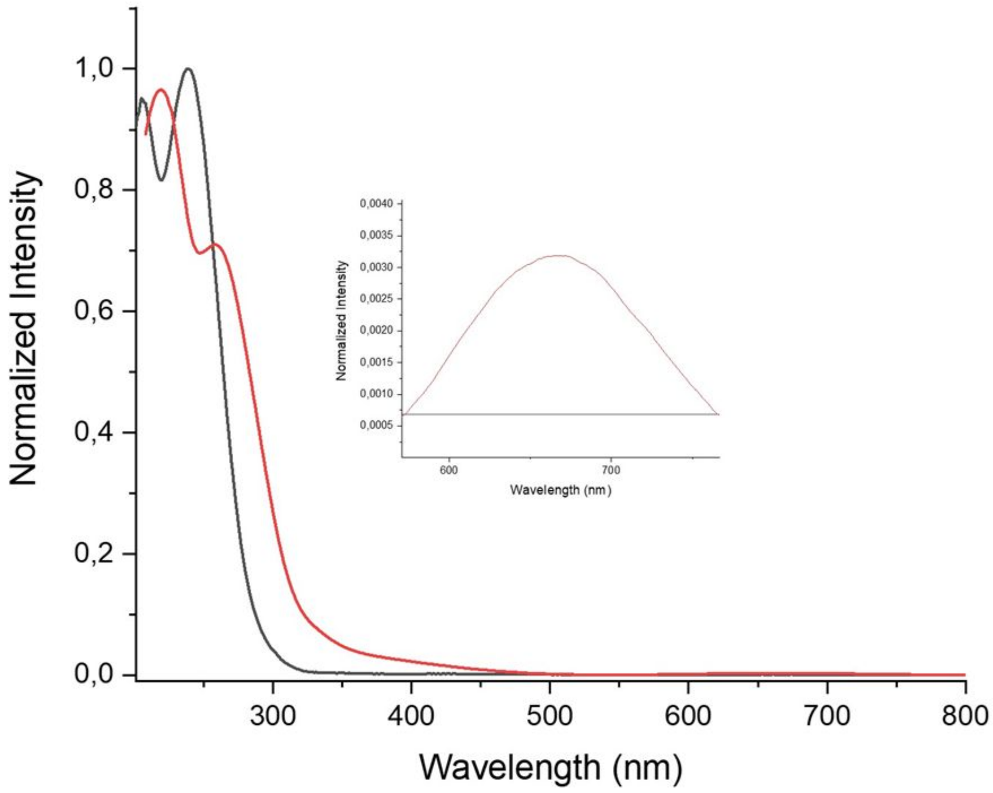

Figure S12: Normalized UV-vis spectra of L (black line) and $[L_2Cu_2(H_2O)_2]\cdot 2(H_2O)$ (red line) in ethanol.

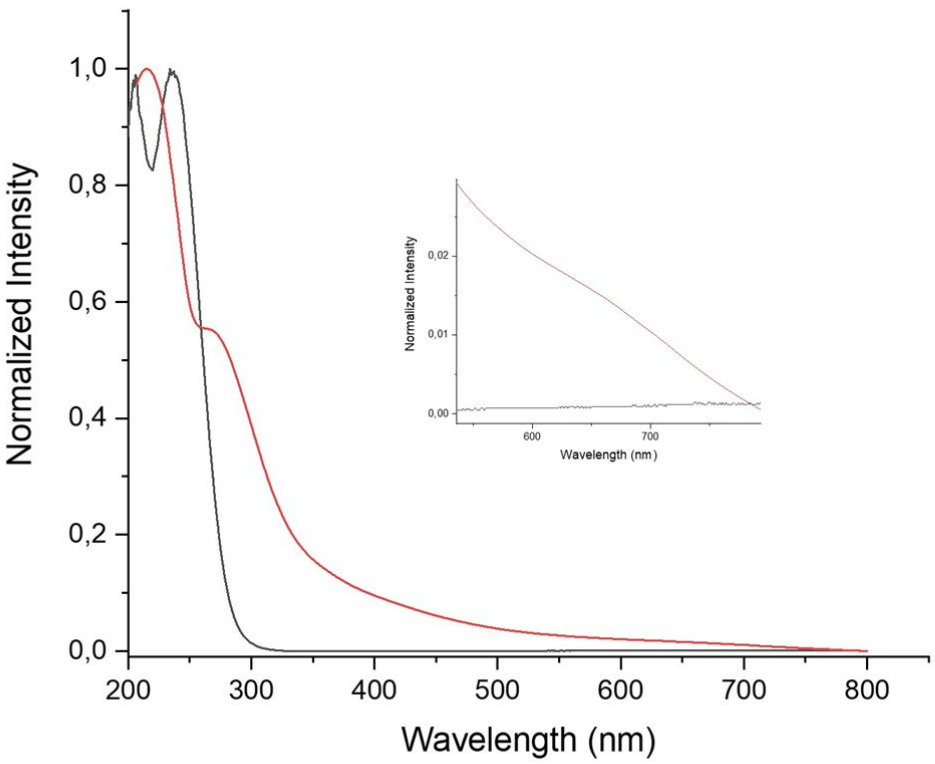

Figure S13: Normalized UV-vis spectra of L (black line) and $[L_2Cu_2(H_2O)_2]\cdot 2(H_2O)$ (red line) in acetonitrile.

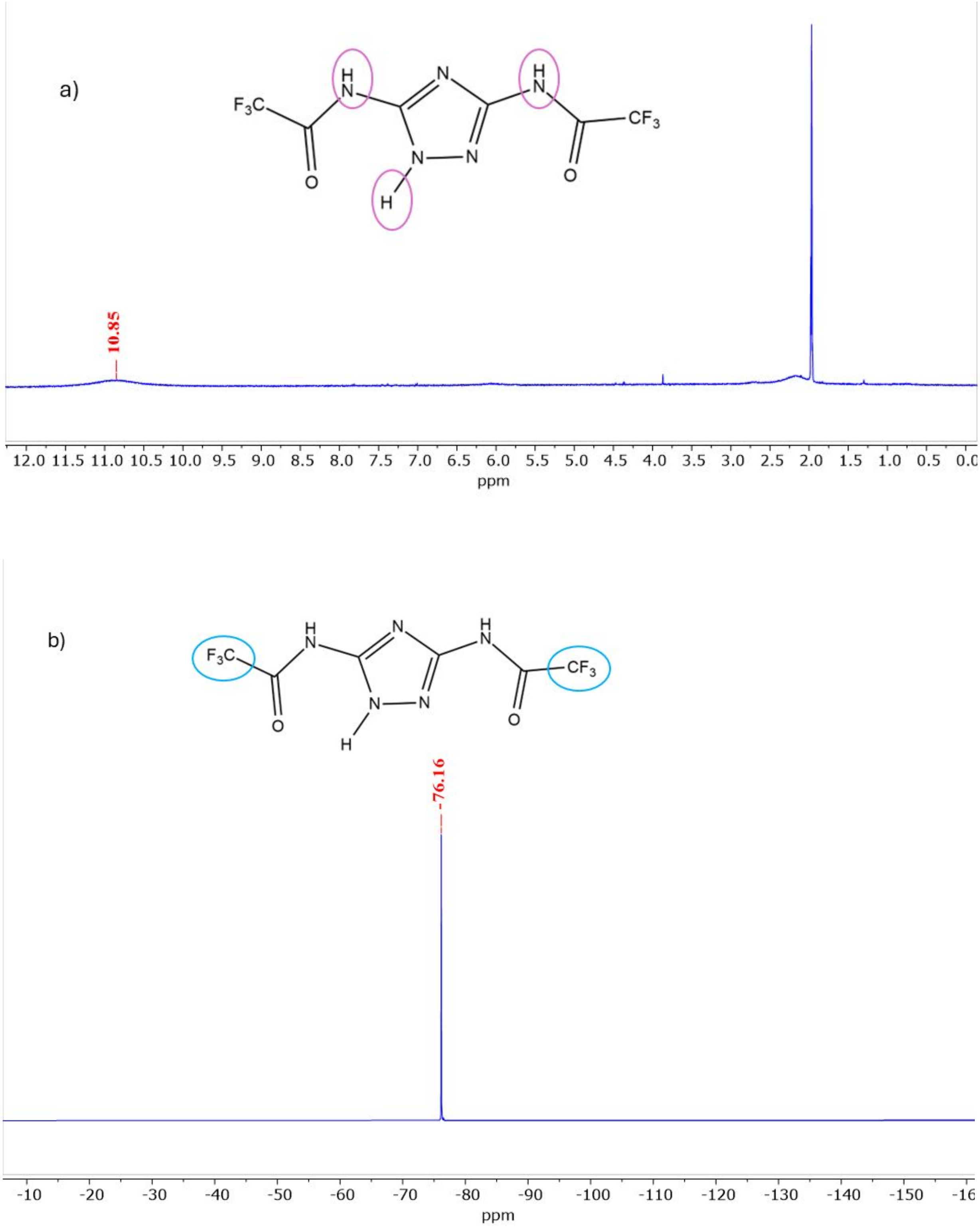

Figure S14: ^{1}H (a) and ^{19}F NMR (b) of ligand L in CD_3CN.

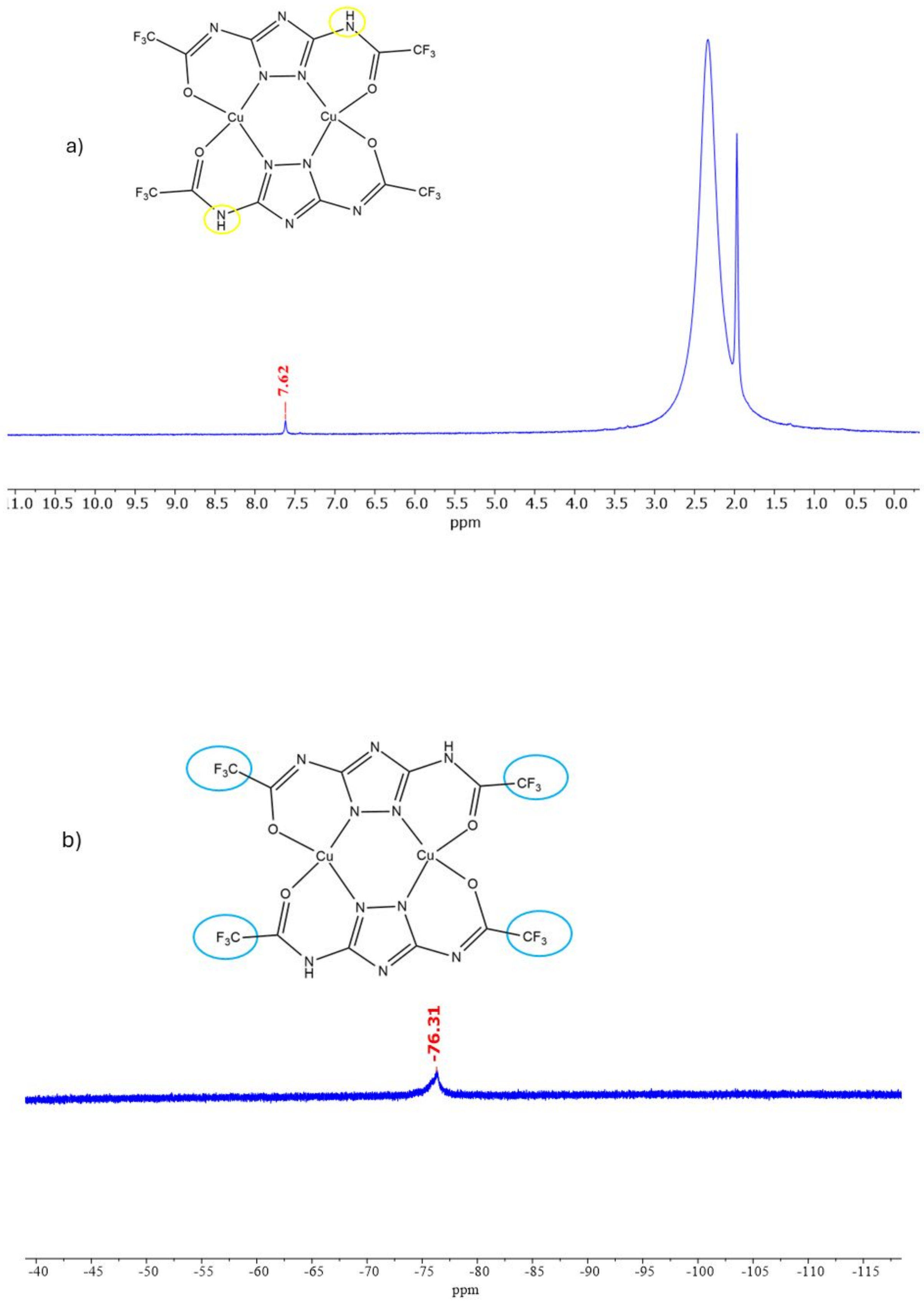

Figure S15. ^{1}H NMR (a) and ^{19}F NMR (b) of $[L_2Cu_2(H_2O)_2]•2(H_2O)$ dissolved in CD_3CN.

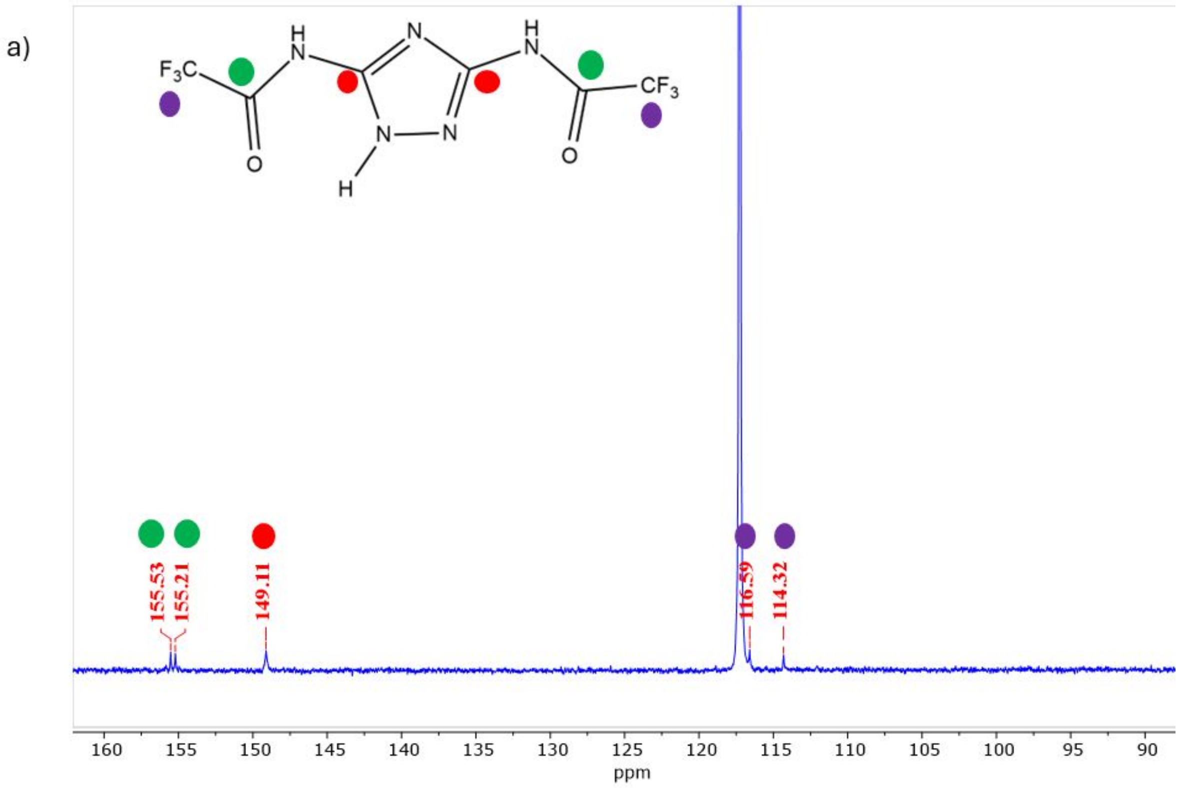

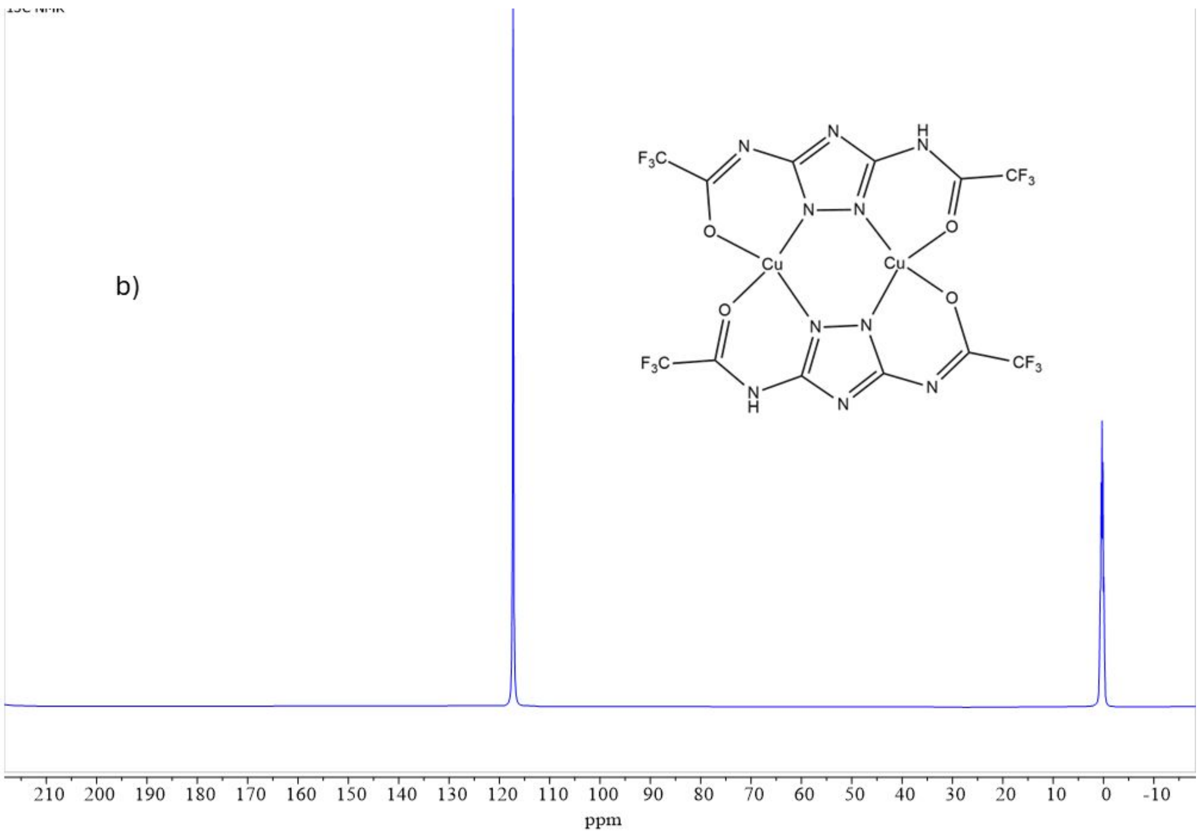

Figure S16. ^{13}C NMR of the free ligand L (a) and ^{13}C NMR (b) of $[L_2Cu_2(H_2O)_2]\cdot 2(H_2O)$, both dissolved in CD_3CN. The ^{13}C NMR of the compound Cu_2L_2 herein reported does not record any signal unless those due to the solvent even though it was obtained after overnight accumulations.

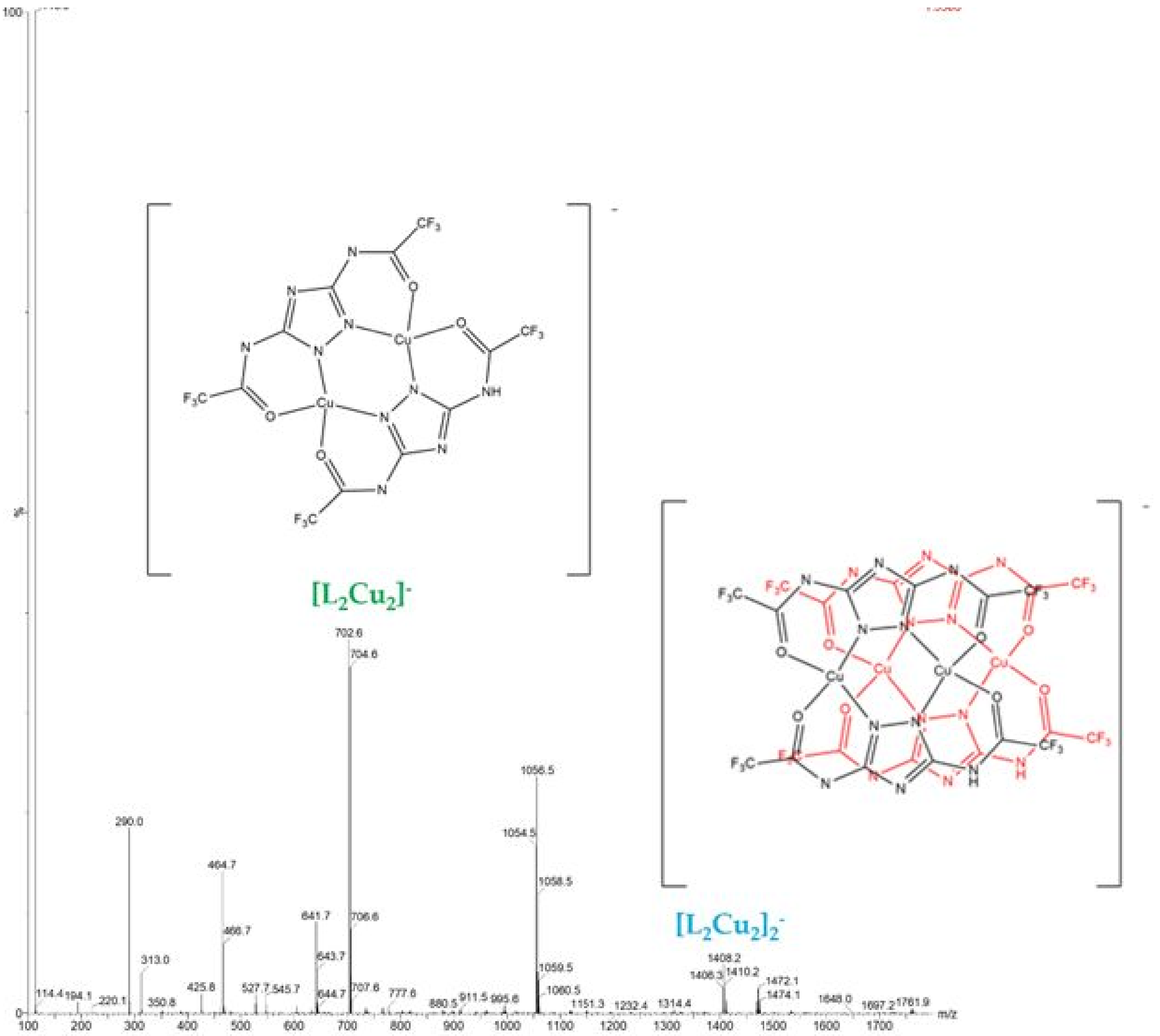

Figure S17: ESI-MS (-) spectrum of $[L_2Cu_2(H_2O)_2]{\bullet}2(H_2O)$ dissolved in EtOH.

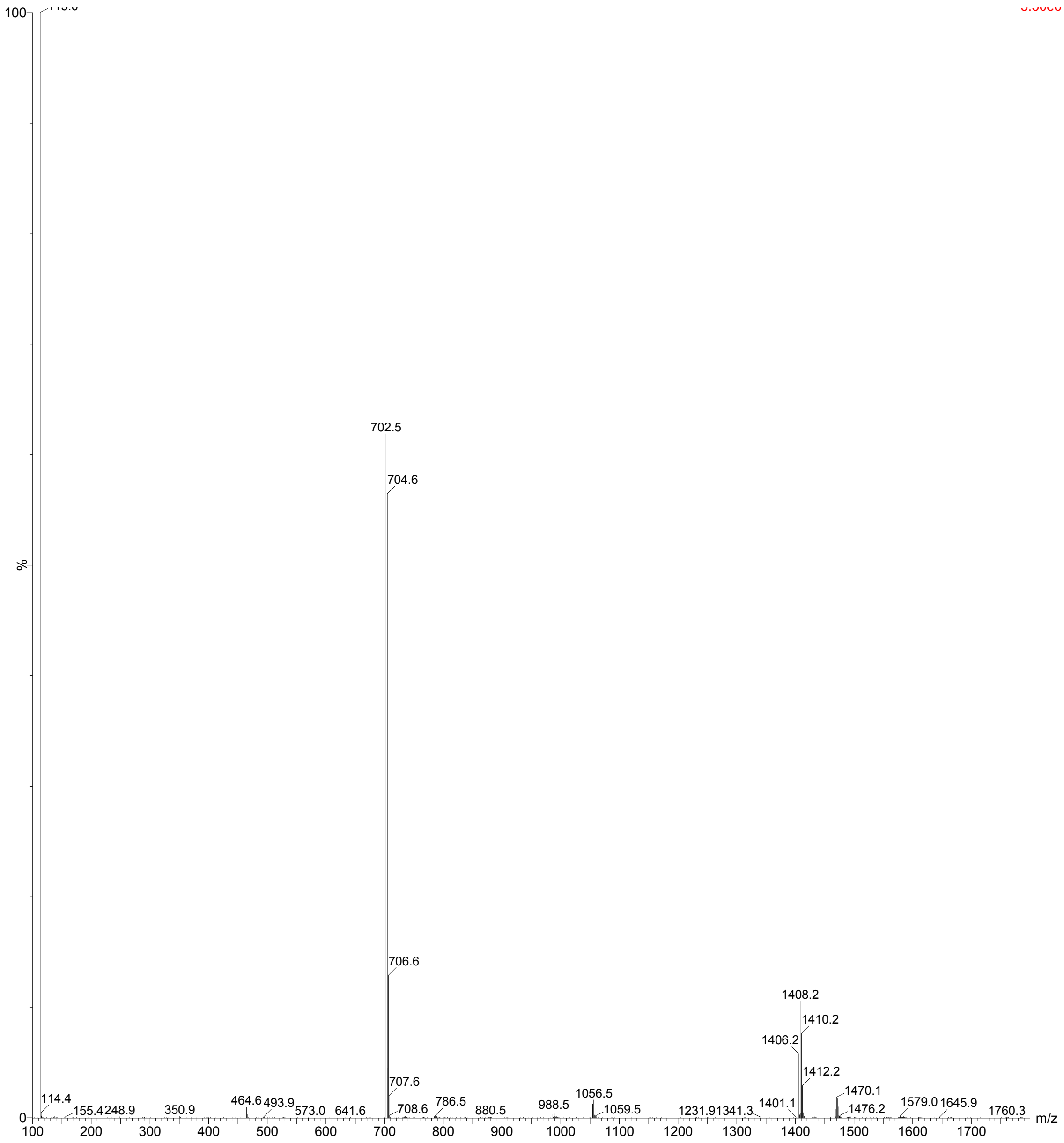

Figure S18. ESI-MS (-) spectrum of $[L_2Cu_2(H_2O)_2]•2(H_2O)$ dissolved in CH_3CN.

UV-Vis Dilution Experiments.

UV-Vis dilution experiments were conducted by dissolving $[L_2Cu_2(H_2O)_2]•2(H_2O)$ in ETOH or CH_3CN and by recording UV-Vis spectra after each dilution within the 2.56-0.005 mM range. The UV-Vis absorbance data at approximately 670 nm and 650 nm for ETOH and CH_3CN, respectively, were fitted using both the dimerization/equal K (EK) and the cooperative CoEK models through an online fitting algorithm available at www.supramolecular.org. The Kd and Ke values represent, respectively, the dimerization and the polymerization constants following the self-aggregation sequence sketched in Scheme 1. The fittings' validity was evaluated by assessing some statistical parameters, the error % on Kd and Ke determinations, and the SSR (Sums of Squares Residuals, the sum of the differences between the predicted value and the mean of the dependent variable). The SSR describes essentially how the curve is well-fitted, and an SSR value approaching zero is meaningful for a better fitting [9]. The result of the fitting is reported in Table S3, while the dilution curves obtained by UV-visible spectroscopy are reported in Figure S10 and Figure 2 (manuscript), and the plots of fitting data obtained in different solvents are in Figures S11 and Figure 2 (manuscript). In Figure S12, the UV-visible spectrum of an ethanol solution of $[L_2Cu_2(H_2O)_2]•2(H_2O)$ is compared to that of the free ligand, and in Figure S13, those recorded in an acetonitrile solution are shown.

$$X + X \xrightleftharpoons{K_2\ (Kd)} X_2$$

$$X_2 + X \xrightleftharpoons{K_3} X_3$$

$$X_3 + X \xrightleftharpoons{K_4} X_4$$

$$X_n + X \xrightleftharpoons{K_i} Xi$$

$$K_d < K_e = K_3 = K_4 ... = K_i$$

Scheme S1. Description of the homogeneous linear aggregation model according to the coEK aggregation – model [9].

Table S3. Ke (and Kd) results, by using the cooperative CoEK model in ethanol and acetonitrile solutions.

**Solvent**	**Kd (M^{-1})**	**Kd Error (%)**	**Ke (M^{-1})**	**Ke Error (%)**	**SSR**
ETOH	1,95 x 10^3	± 0,62	3,89 x 10^3	± 1,24	1.50 x 10^{-3}
CH_3CN	4.00 x 10^2	± 0,38	8.01 x 10^2	± 0.38	1.77 x 10^{-2}

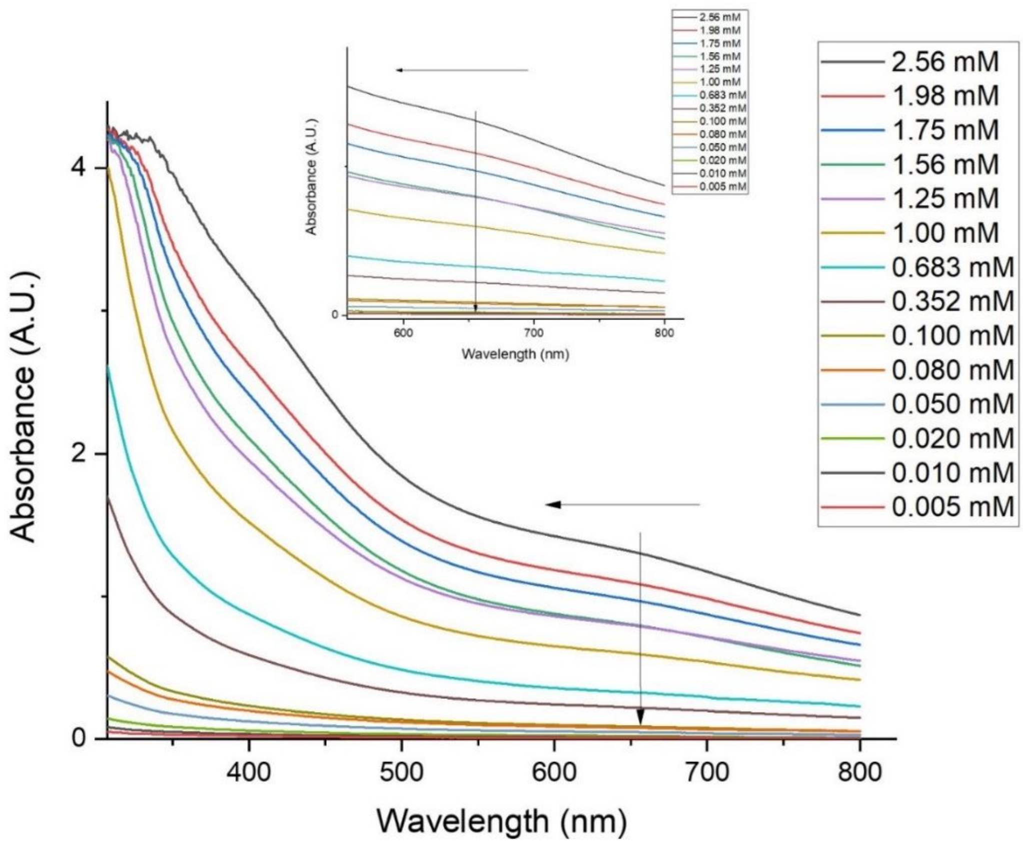

Figure S19. UV-Vis dilution experiment of $[L_2Cu_2(H_2O)_2]\bullet 2(H_2O)$ in CH_3CN. The samples were prepared in series with an aliquot of the most concentrated solution undergoing serial dilution from 2.56 mM to 0.006 mM. In the inset the enlargement of the 600-800 nm region.

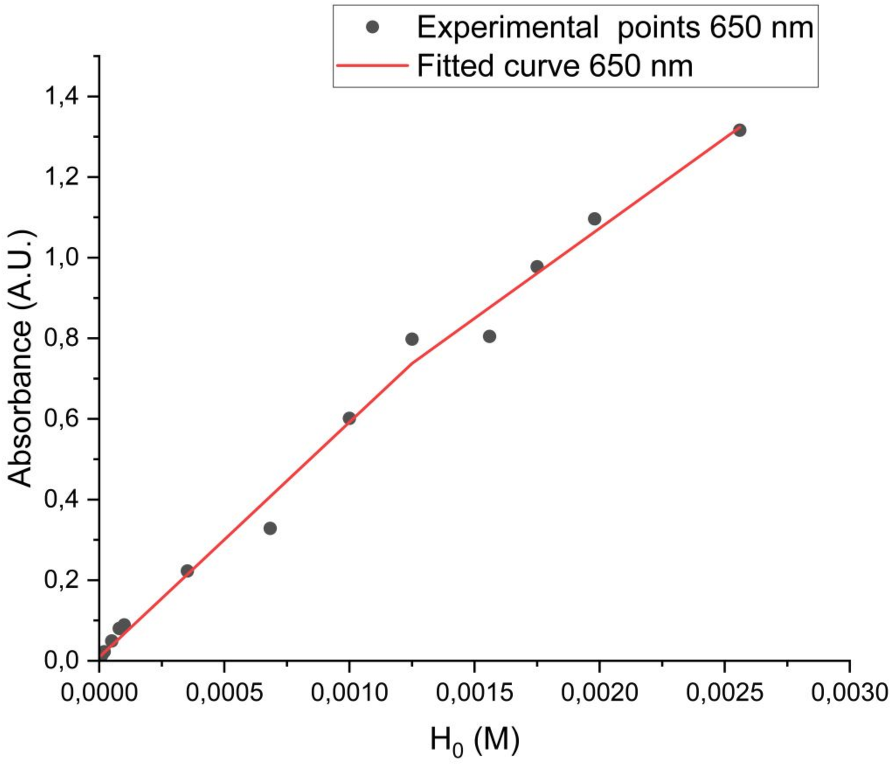

Figure S20: Data fitting of UV-vis dilution experiment of $[L_2Cu_2(H_2O)_2]\bullet 2(H_2O)$ in CH_3CN, by using an algorithm embedded in www.supramolecular.org.

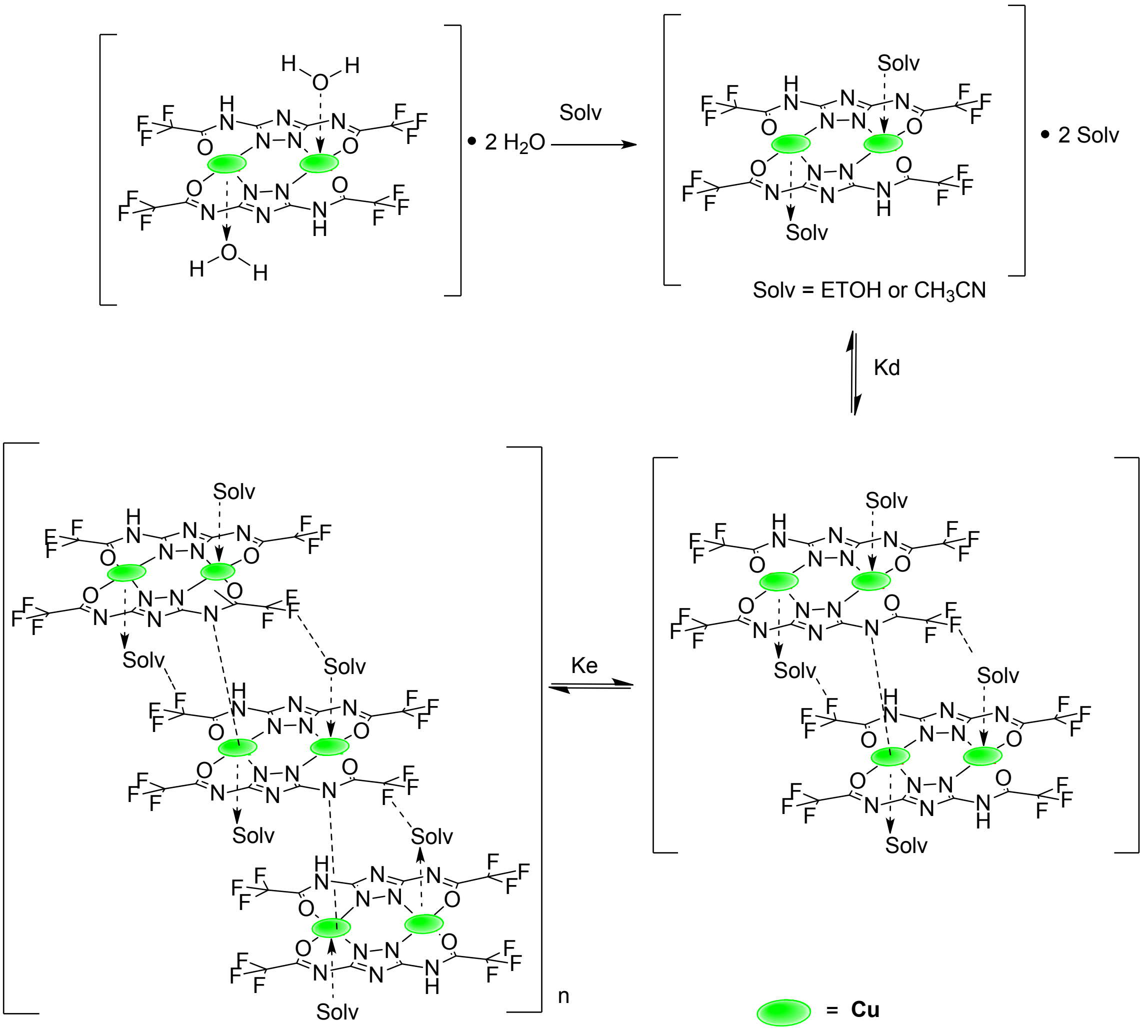

Scheme S2. Representation of the proposed $[L_2Cu_2(H_2O)_2]$•$2(H_2O)$ self-association equilibria in ethanol or acetonitrile solution.

Catalysis

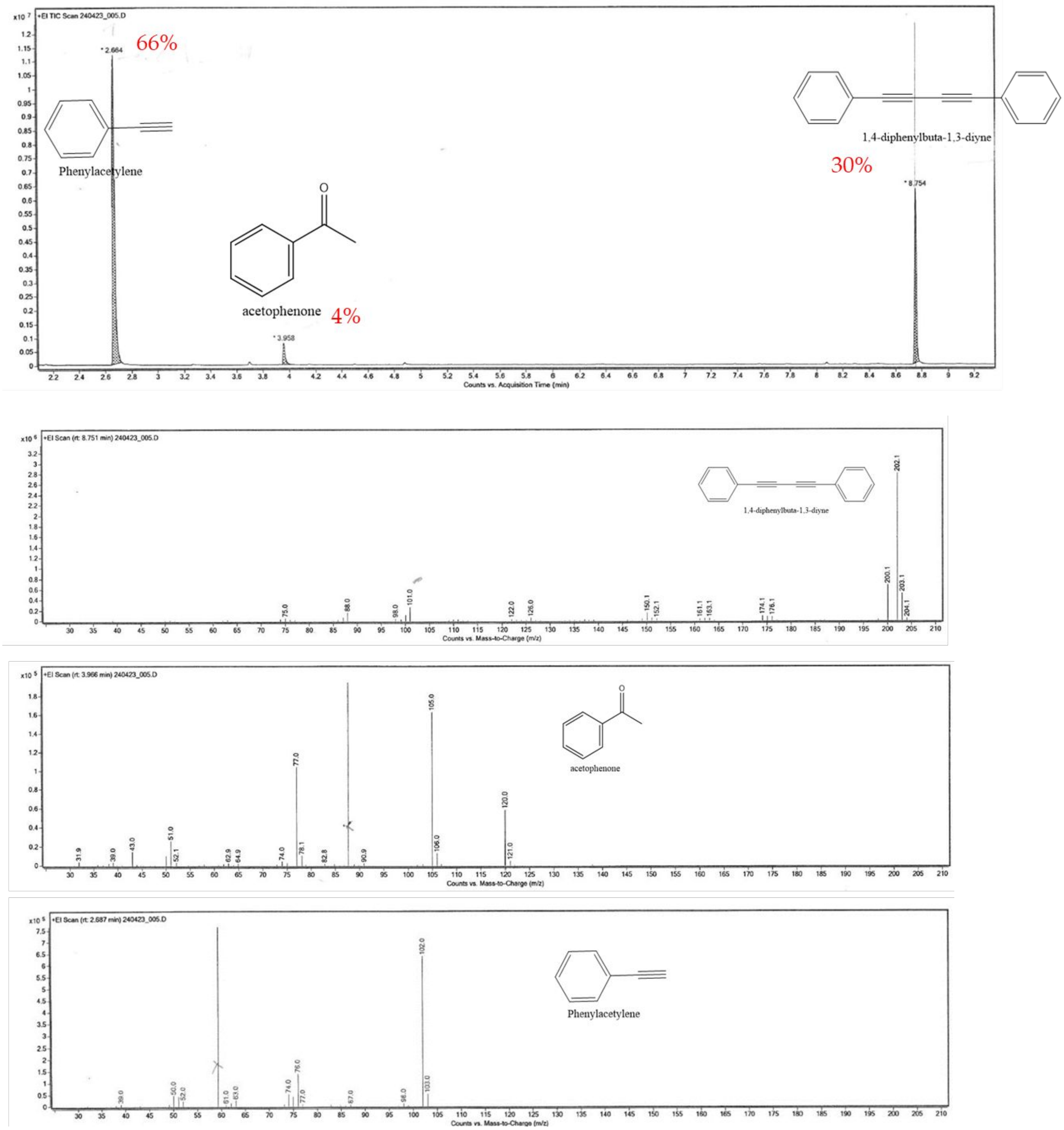

Figure S21. GC-MS chromatogram (top) and EI-MS spectra (bottom, following the inset molecular structures) of phenylacetylene, acetophenone, and **1,4-diphenylbutan-1,3-diyne**.

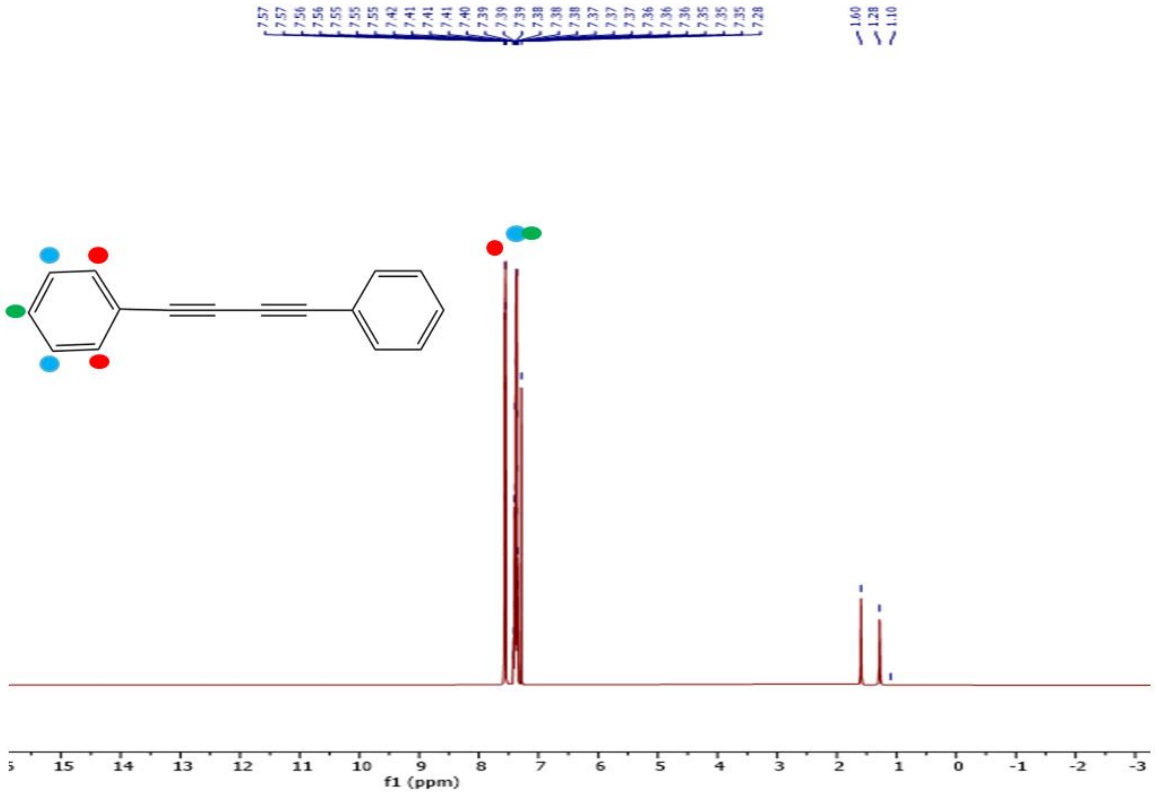

Figure S22: ^{1}H NMR (recorded at 500 MHz) of 1,4-diphenylbutadiyne in $CDCl_3$ obtained after filtration over a pad of silica gel.

Table S4. List of results in terms of catalyzed phenylacetylene homocoupling reactions from the literature.

Reaction scheme	Catalyst, reaction conditions	Yield %	References
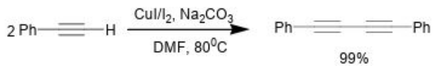	CuI, I_2 and a solid base Na_2CO_3 in DMF at 80 °C	99	[10]
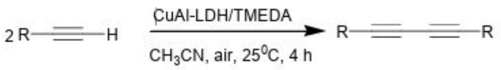	CuAl–LDH Copper (obtained by coprecipitation from copper and alluminium nitrate, LDH are Layered Double Hydroxide), acetonitrile, aria, 25°c, 4 hours	R = CH_2OH 86%<br>R = CH_2CH_2OH 80%<br>R = n-C_4H_9 84%<br>R =$Si(CH_3)_3$ 74%<br>R = CH_2OCOPh 82%	[11]
$Pd(OAc)_2/PCy_3$	$K_3PO4.3H_2O$ as base in	65-98	[12]

	toluene at 80°C for only 10-30 min		
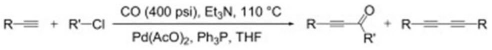	Piperidine, Et_3N, K_2CO_3, Reactions were performed under O_2 at 25 °C for 4 h; catalyst: 5 mol-%.	98 (piperidine), 66 (Et_3N), 60 (K_2CO_3)	[13]
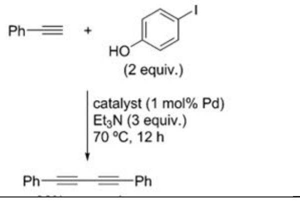	Mesoporous-Silica Supported Palladium	86	[14]

References

[1] APEX3 Software Package V2019; Bruker AXS Inc.: Madison, WI, **2019**.

[2] Bruker SAINT, v8.40A: Part of the APEX3 Software Package V2019; Bruker AXS Inc.: Madison, WI, **2019**.

[3] Bruker SADABS V2016/2: Part of the APEX3 Software Package V2019; Bruker AXS Inc.: Madison, WI, **2019**.

[4] Sheldrick, G. M. SHELXT − Integrated Space-Group and Crystal-Structure Determination. Acta Crystallogr., Sect. A: Found. Adv.**2015**, 71, 3−8, doi: 10.1107/S2053273314026370.

[5] Sheldrick, G. M. Crystal Structure Refinement with SHELXL. Acta Crystallogr., Sect. C: Cryst. Struct. Commun. **2015**, 71, 3−8.

[6] Macrae, C. F.; Bruno, I. J.; Chisholm, J. A.; Edgington, P. R.; McCabe, P.; Pidcock, E.; Rodriguez-Monge, L.; Taylor, R.; Streek, J.; van de Wood, P. A. Mercury CSD 2.0 − New Features for the Visualization and Investigation of Crystal Structures. J. Appl. Crystallogr. **2008**, 41, 466−470, doi: 10.1107/S0021889807067908

[7] Di Nicola, C.; Marchetti, F.; Tombesi, A.; Xhafa, S.; Campitelli, P.; Moroni, M.; Galli, S.; Pettinari, R.; Pettinari, C. Antibacterial Activity of Copper Pyrazolate Coordination Polymers. *New Journal of Chemistry* **2023**, *47*, 19047–19056, doi:10.1039/d3nj02378h.

[8] Dias Selassie, C.; Lien, E.J.; Khwaja, T.A. Synthesis and Evaluation of Guanazole Prodrugs as Antineoplastic Agents; J Pharm Sci, **1981**; Vol. 5, 1281-1283, doi: 10.1002/jps.2600701126.

[9] Von Krbek, L.K.S.; Schalley, C.A.; Thordarson, P. Assessing Cooperativity in Supramolecular Systems. Chem Soc Rev **2017**, 46, 2622–2637, doi:10.1039/C7CS00063D.

[10] D. Li, K. Yin, J. Li and X. Jia, *Tetrahedron Lett*., **2008**, *49*, 5918 5919.

[11] B. C. Zhu and X. Z. Jiang, *Appl. Organometal. Chem.,* **2007**, *21*, 345 349.

[12] Ma, Xiaowei; Liu, Yan; Liu, Ping; Xie, Jianwei; Dai, Bin; Liu, Zhiyong. *Appl Organomet Chem* **2013**, *27*, 707.

[13] S. Srivastava, A. Ali, A. Tyagi and R. Gupta, *Eur. J. Inorg. Chem*. **2014**, 21132123.

[14] Francisco Alonso and Miguel Yus. Heterogeneous Catalytic Homocoupling of Terminal Alkynes *ACS Catalysis* **2012,** *2*, 1441-1451. DOI: 10.1021/cs300195r
